# Synthesis, Structure, Electrochemical Properties, and Antioxidant Activity of Organogermanium(IV) Catecholate Complexes

**DOI:** 10.3390/ijms25169011

**Published:** 2024-08-19

**Authors:** Daria A. Burmistrova, Nadezhda P. Pomortseva, Yulia K. Voronina, Mikhail A. Kiskin, Fedor M. Dolgushin, Nadezhda T. Berberova, Igor L. Eremenko, Andrey I. Poddel’sky, Ivan V. Smolyaninov

**Affiliations:** 1 Chemistry Department, Astrakhan State Technical University, 16 Tatisheva Str., 414056 Astrakhan, Russia; d.burmistrova@astu.org (D.A.B.); ogorodnikova1503@rambler.ru (N.P.P.); nberberova@astu.org (N.T.B.); 2 N.S. Kurnakov Institute of General and Inorganic Chemistry, Russian Academy of Sciences, 119991 Moscow, Russia; juliavoronina@mail.ru (Y.K.V.); mkiskin@igic.ras.ru (M.A.K.); fmdolgushin@gmail.com (F.M.D.); ilerem@igic.ras.ru (I.L.E.); 3 Institute of Inorganic Chemistry, University of Tübingen, Auf der Morgenstelle 18, 72076 Tübingen, Germany

**Keywords:** redox-active ligand, germanium, catecholate, X-ray, CV, radical scavenging activity

## Abstract

A series of novel organogermanium(IV) catecholates **1**–**9** of the general formula R’_2_Ge(Cat), where R’ = Ph, Et, have been synthesized. Compounds were characterized by ^1^H, ^13^C NMR, IR spectroscopy, and elemental analysis. The molecular structures of **1**–**3**, **6**, and **8** in crystal state were established using single-crystal X-ray analysis. The complexes are tetracoordinate germanium(IV) compounds containing a dioxolene ligand in a dianion (catecholato) form. Electrochemical transformations of target germanium(IV) complexes have been studied by cyclic voltammetry. The electro-oxidation mechanism of complexes **1**–**5**, **7**, and **10** (the related complex Ph_2_Ge(3,5-Cat) where 3,5-Cat is 3,5-di-*tert*-butylcatecholate) involves the consecutive formation of mono- and dicationic derivatives containing the oxidized forms of redox-active ligands. The stability of the generated monocations depends both on the hydrocarbon groups at the germanium atom and on the substituents in the catecholate ring. Compounds **6**, **8**, and **9** are oxidized irreversibly under the electrochemical conditions with the formation of unstable complexes. The radical scavenging activity and antioxidant properties of new complexes were estimated in the reaction with DPPH radical, ABTS radical cation, and CUPRAC_TEAC_ assay. It has been found that compounds **8** and **9** with benzothiazole or phenol fragments are more active in DPPH test. The presence of electron-rich moieties in the catecholate ligand makes complexes **5** and **7**–**9** more reactive to ABTS radical cation. The value of CUPRAC_TEAC_ for organogermanium(IV) catecholates varies from 0.23 to 1.45. The effect of compounds **1**–**9** in the process of lipid peroxidation of rat liver (Wistar) homogenate was determined in vitro. It was found that most compounds are characterized by pronounced antioxidant activity. A feature of complexes **1**, **3**, and **5**–**9** is the intensification of the antioxidant action with the incubation time. In the presence of additives of complexes **3**, **5**, **6**, and **8**, an induction period was observed during the process of lipid peroxidation.

## 1. Introduction

Germanium(II/IV) complexes attract the special attention of researchers due to the wide coordination capabilities of the germanium atom, in particular, the ability to form hypercoordinated compounds as well as the manifestation of catalytic activity [1,2]. Currently, a fairly large number of studies are devoted to the synthesis and study of the molecular and electronic structure of germylenes, which have significant potential to be used in various fields of chemistry [3,4,5,6]. Catalysis using germanium(II/IV) derivatives is one of the practically significant research areas aimed at finding alternatives to the use of transition metals. The application of redox-active ligands in combination with a non-transition element opens up prospects for the design of substances with expanded valence capabilities and unusual reactivity. Such compounds can serve as surrogates for transition metals in the catalytic cycle, participating in electron, proton, and functional group transfer reactions [7].

As a result, a certain number of works are devoted to the preparation of germylenes containing redox-active ligands [8,9,10,11,12,13] and the study of their reactivity and catalytic properties [14,15,16,17,18]. However, germanium(IV) coordination compounds with redox-active ligands are also being actively studied [19,20,21,22,23,24,25,26]. These compounds have potential in catalytic transformations [27,28] and can be used as a basis for developing new functional materials, e.g., lithium-ion batteries [29,30]. Among the recently obtained germanium(IV) compounds, bis-ligand complexes with dioxolene ligands containing various substituents turned out to be the most promising from a practical point of view. This is due to their catalytic activity in the reaction of dimerization of arylated alkenes, oligomerization of alkenes, hydrosilylation of aldehydes, hydroboration of alkynes, and Friedel–Crafts alkylation of indole, diphenylamine, or anisole [31,32,33].

Another important aspect of using germanium(II/IV) coordination and organometallic derivatives is their biological activity. Germanium compounds are characterized by various physiological properties: anti-inflammatory, antiviral, immunostimulating, antibacterial, and antitumor [34,35,36,37,38]. Comprehensively studied germanium derivatives are germanium 2-carboxyethyl sesquioxide (Ge-132), which in aqueous media is 3-(trihydroxygermyl)-propanoic acid, as well as spirogermanium derivatives, germatranes, and germylporphyrins. Currently, there is great interest in an approach based on the combination of a metal-containing center and natural or synthetic pharmacologically active ligands in one molecule [39,40].

To date, germanium(IV) compounds containing well-known biologically active fragments, such as ascorbic acid, crysine, quercetin, dihydroartemisinin, dihydroxycoumarine, adrenaline, and steroids, have been obtained [41,42,43,44,45,46,47,48]. The Ge-132 compound [49], as well as germanium(IV) complexes with ascorbic acid or polyphenols, exhibit pronounced antioxidant activity. As mentioned above, germanium(IV) bis-catecholate compounds have been better studied, while there are only a few works devoted to organogermanium(IV) monocatecholate derivatives [50,51]. Therefore, the purpose of this paper was to obtain new organogermanium(IV) complexes based on the sterically hindered catechols or catechol thioethers, containing different groups at the sulfur atom, to establish the molecular structure to study the electrochemical transformations and radical scavenging properties in the reaction with DPPH radical or ABTS radical cation, in CUPRAC assay, in the process of lipid peroxidation in vitro.

## 2. Results and Discussion

### 2.1. Synthesis

**Scheme 1:**
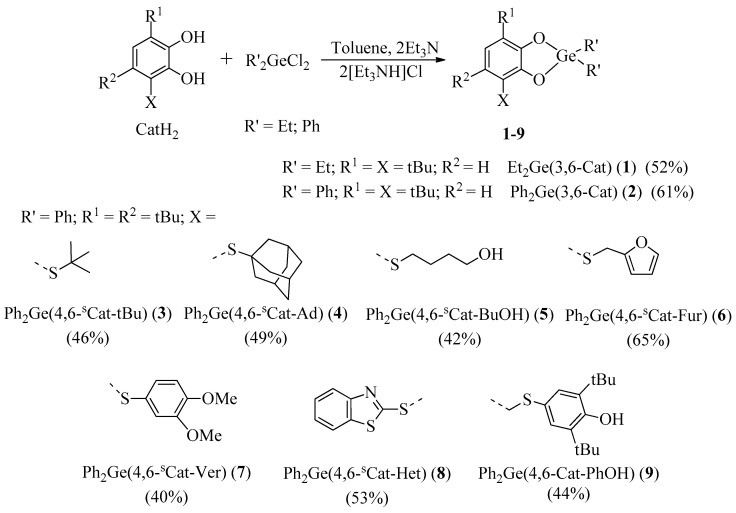
Synthesis of diorganogermanium(IV) catecholates.

Organogermanium(IV) complexes **1**–**9** with catecholate ligands of the general formula R’_2_GeCat were synthesized in the exchange reaction between corresponding catechols or catechol thioethers and R’_2_GeCl_2_ in equimolar ratio in toluene in the presence of two equivalents of triethylamine under anaerobic conditions (Scheme 1).

Complex Ph_2_Ge(3,5-Cat) (**10**) with 3,5-di-*tert*-butylcatecholate was prepared according to the previously described method [50]. The structures of the complexes were confirmed by the data of ^1^H and ^13^C{^1^H} NMR- (Figures S1–S18), FT-IR spectroscopy, elemental analysis, and HRMS. Complexes **1**–**9** were isolated during filtration on air as colorless or white crystalline powders with a yield of up to 65%.

The ^1^H and ^13^C{^1^H} NMR spectra of **1**–**9** confirm the composition of compounds and contain sets of peaks from protons (and carbons, respectively) of all functional groups in structures of these complexes. For example, two equivalent aromatic protons in 3,6-Cat in **1** and **2** give rise to singlets at 6.66 and 6.77 ppm, respectively; the singlets from aromatic single protons in 3-substituted 4,6-di-*tert*-butylcatecholates in **3**–**9** are downfield shifted to the region 6.83–7.02 ppm.

### 2.2. X-ray Structure

#### 2.2.1. The Molecular Structures in the Crystal State

**Figure 1:**
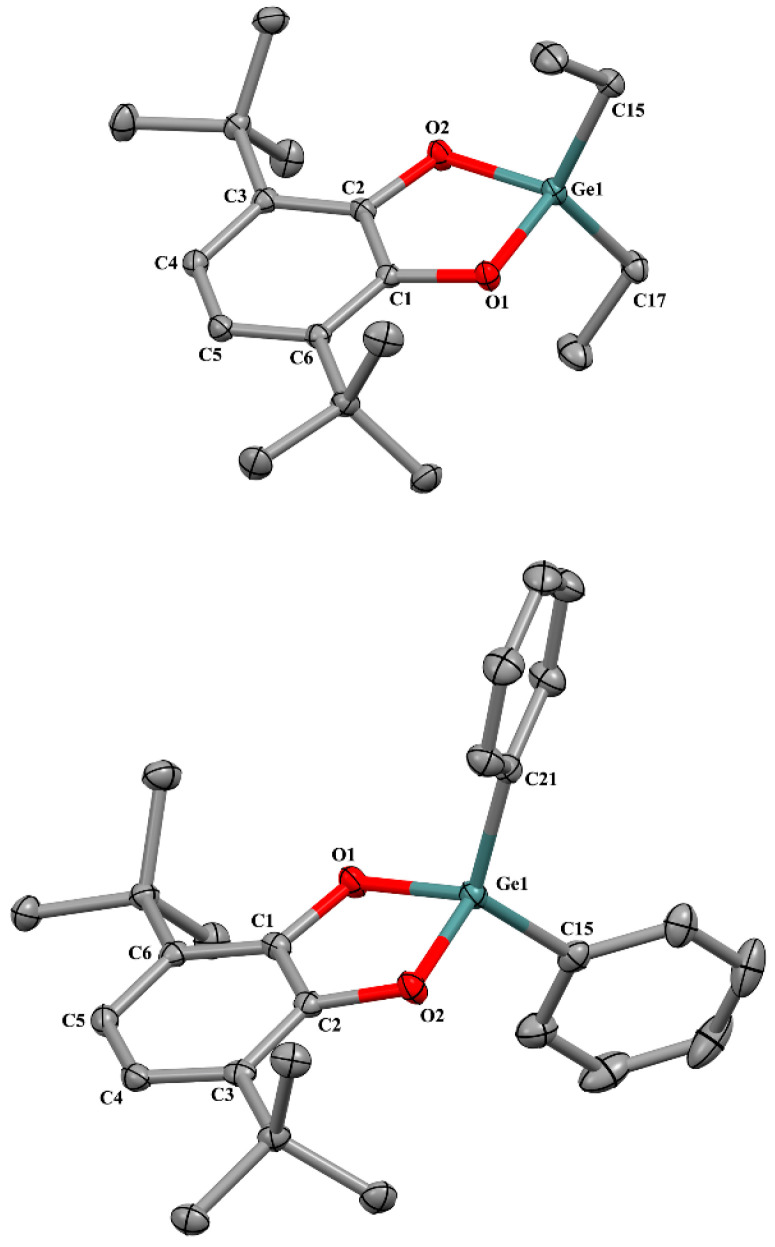
X-ray structures of Et_2_Ge(3,6-Cat) **1** (top) and Ph_2_Ge(3,6-Cat) **2** (bottom). The hydrogen atoms are omitted for clarity. The ellipsoids are of 50% probability.

**Figure 2:**
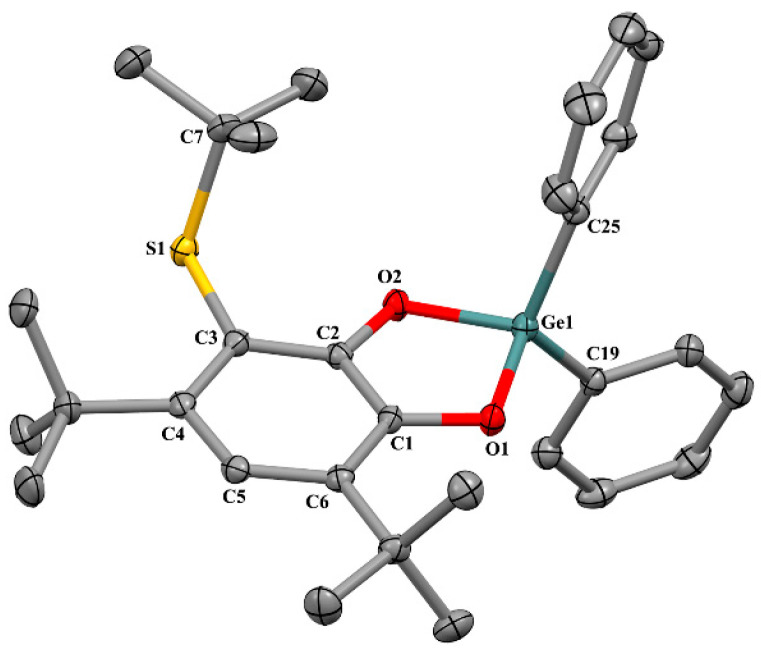
X-ray structure of Ph_2_Ge(4,6-^s^Cat-tBu) **3**. The hydrogen atoms are omitted for clarity. The ellipsoids are of 50% probability.

**Figure 3:**
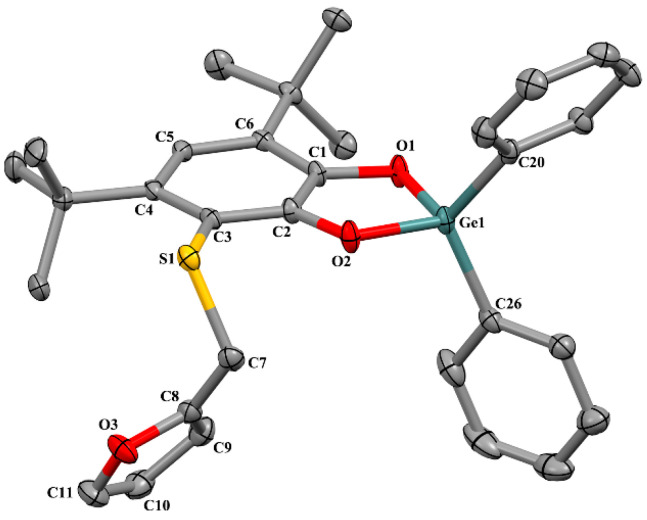
X-ray structure of Ph_2_Ge(4,6-^s^Cat-Fur) **6**. The hydrogen atoms are omitted for clarity. The ellipsoids are of 50% probability.

**Figure 4:**
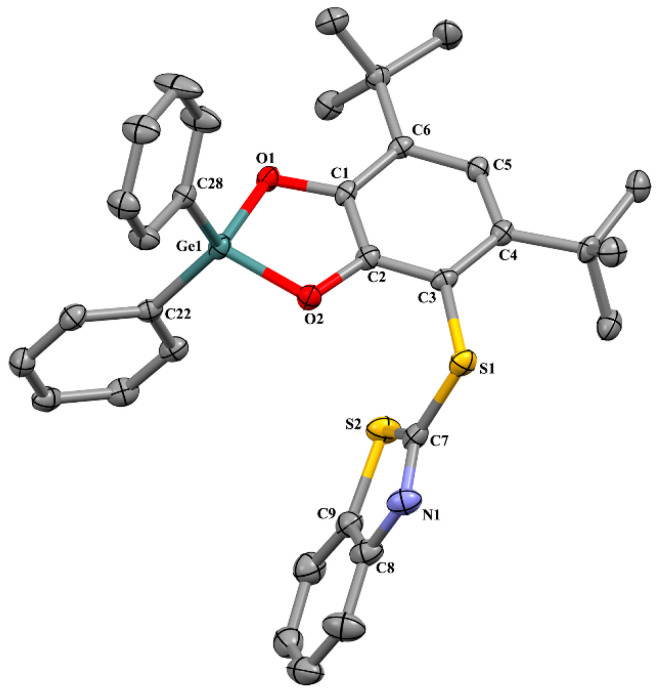
X-ray structure of Ph_2_Ge(4,6-^s^Cat-Het) **8**. The hydrogen atoms are omitted for clarity. The ellipsoids are of 50% probability.

Crystals of **1**–**3**, **6**, and **8** suitable for single-crystal X-ray analysis were grown from solutions in n-hexane. The details of X-ray experiments and structure determination are given in Table S1. The X-ray structures are shown in Figure 1, Figure 2, Figure 3 and Figure 4; the selected structural data are given in Table 1. The unit cells of **2** and **8** contain two independent molecules of complexes with very close structural characteristics (see Table 1).

The common feature of these complexes is a slightly distorted tetrahedral coordination environment of germanium atom Ge1 irrespective of the presence of potential coordinating functions in the catecholate structure (e.g., furan-2-yl in **6** or benzothiazole-2-yl in **8**). The tetrahedral environment of Ge1 is formed by oxygen atoms O1 and O2 of chelating ligand and carbon atoms of ethyl (in **1**) or phenyl (**2**, **3**, **6**, **8**) groups. The geometric parameter for four-coordinate compounds, *τ*_4_, proposed by R.P. Houser et al. (*τ*_4_ = 1.00 for a perfect tetrahedral geometry, and *τ*_4_ = 0.00 for a perfect square planar geometry) [52] was calculated to be 0.99 for **1**, 0.91 and 0.89 for two independent molecules of **2**, 0.91 for **3**, 0.91 for **6**, and 0.92 for both independent molecules of **8**. 

The Ge1–O1 and Ge1–O2 bonds in the complexes studied are in the range of 1.80–1.82 Å, which correspond to germanium(IV) monocatecholate complexes with coordination number 4. For example, the Ge–O bonds are 1.80–1.82 Å in (3,5-Cat)GeL where L is N,P-chelating ligand [53] and 1.79–1.84 Å in R_2_Ge(3,5-Cat) where R is 2,4,6-tri-isopropylphenyl [54] or (4-methylbenzene-1-sulfonyl)(triphenylphosphonio)methanidyl [55]. In the related hexacoordinated germanium(IV) bis-catecholates, such Ge–O bonds are longer and vary in the range of 1.85–1.90 Å [24,31,56,57,58,59,60,61,62,63,64,65].

The bonds O1–C1 and O2–C2 for all complexes are in the range of 1.376–1.387 and 1.365–1.388 Å, respectively, and correspond to ordinary O–C bonds (1.34–1.39 Å) in different catecholate complexes [62,66,67,68,69,70,71,72,73]. The six-membered carbon cycles C (1–6) in complexes are aromatic with C–C_av_ distances of 1.400 ± 0.008 (**1**), 1.397 ± 0.010 (**2**), 1.406 ± 0.018 (**3**), 1.400 ± 0.016 (**6**), and 1.401 ± 0.023 (**8**) Å, respectively. Worthy of note, going from symmetrical 3,6-di-*tert*-butylcatecholate (in **1** or **2**) to non-symmetric 4,6-di-*tert*-butylcatecholate (in **3**, **6**, or **8**), a more pronounced alternation of C–C relations is observed, which is pointing in an increase in deviation from the average value C–C_av_. The X-ray data on the related diorganylgermanium(IV) catecholate complexes of the type (3,5-Cat)GeR_2_ found in CCDC, where R is 2,4,6-tri-isopropylphenyl or (4-methylbenzene-1-sulfonyl)(triphenylphosphonio)methanidyl, show that the corresponding bond lengths O1–C1, O2–C2, and C–C_av_ are 1.390, 1.436, and 1.403 ± 0.015 Å [54] or 1.344, 1.374, and 1.397 ± 0.015 Å [55], respectively.

#### 2.2.2. The Features of Crystal Packing

**Figure 5:**
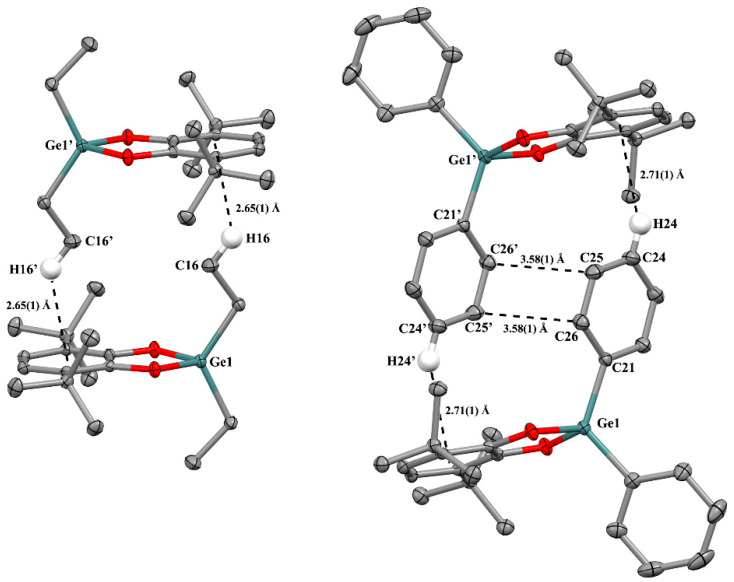
The formation of dimers in crystals of **1** (left) and **2** (right).

The organization of the crystal packing in complexes **1**, **2**, **3**, **6**, and **8** differs remarkably and depends on the nature of functional groups in these compounds. The germanium complexes **1** and **2** with 3,6-di-*tert*-butylcatecholate form dimers due to the short contacts of T-type between the organic group at germanium atom and π-system of catecholate: the contact C16–H16…π-system C(1′-6′) is 2.65(1) Å for diethylgermanium complex **1**, and the contact C24–H24…π-system C(1′-6′) is 2.71(1) Å (Figure 5).

The distances C16–H16…C1′-C6′ in **1** vary in the range of 2.84–3.17(1) Å, and C24–H24…C1′-C6′- in the range of 2.94–3.26(1) Å, which is close to the analogical C–H…π-system interactions (<3.05 Å) discussed by Nishio [74]. Additionally, there are short contacts between carbon atoms C25 and C26 of two phenyl rings C(21–26) in such dimers in diphenylgermanium complex **2**: the distances C25…C26′ and C26…C25′ are 3.58(1) Å, which is close to the sum of van-der-Waals radii of carbon atoms (1.85 Å [75]).

**Figure 6:**
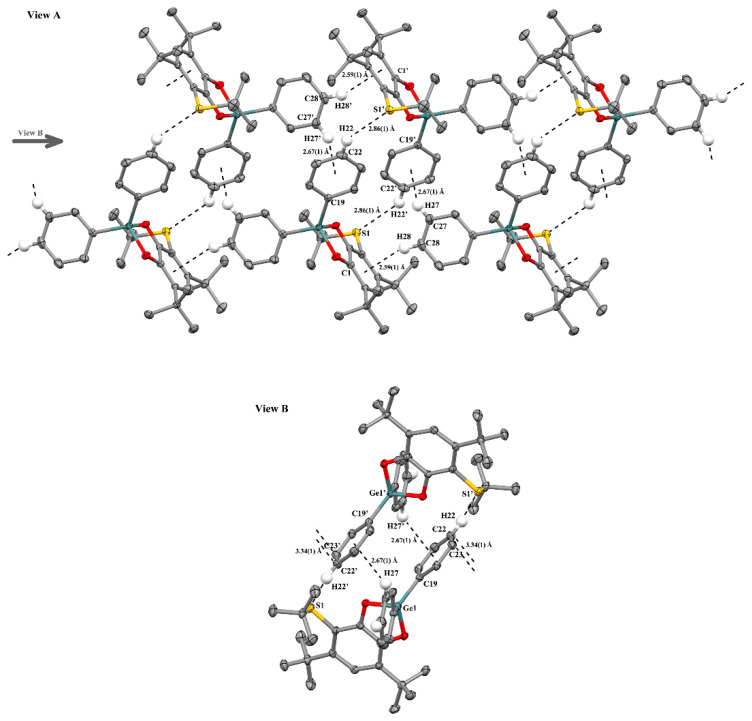
The intermolecular interactions in crystal of **3**. Direction of View B is shown by arrow.

Complex **3** with 3-*tert*-butylthio-4,6-di-*tert*-butylcatecholate in crystalline state forms linear chains due to short contacts of T-type between the phenyl group C(25–30) of one molecule and the π-system of the ring C(1′-6′) of the next molecule (Figure 6): the contact C28–H28…π-system C(1′-6′) is 2.59(1) Å (the corresponding distances C28–H28…C1′-C6′ are 2.84–3.15(1) Å). These chains are connected by the short contacts C27–H27…π-system C(19′-24′) of 2.67(1) Å and by the short contacts C22–H22…S(1′) of 2.86(1) Å. Also, there is interaction between phenyl rings C(19–24) of neighboring molecules with the distances C22…C23′ and C23…C22′ of 3.34(1) Å.

**Figure 7:**
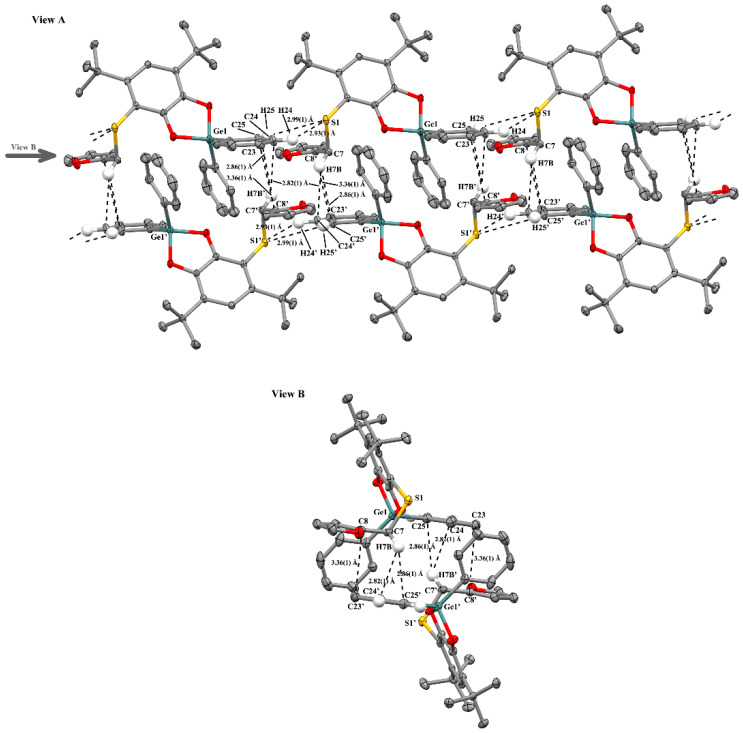
The intermolecular interactions in crystal of **6**. Direction of View B is shown by arrow.

The crystal composition of furan-2-yl-containing complex **6** is more complicated. Molecules of **6** also form chains, however, via the short contacts between phenyl group carbons of one molecule and the sulfur atom of the next molecule (Figure 7): the distances C24–H24…S(1′) and C25–H25…S(1′) are 2.93(1) Å and 2.99(1) Å, respectively. These chains are connected through the interactions between the C–H bond of the methylene group and carbon atoms C24′ and C25′ of phenyl in the neighboring chain: the distances C7–H7B…C24′ is 2.82(1) Å and C7–H7B…C25′ is 2.86(1) Å, as well as through contacts between carbon atoms C8 of furan-2-yl group of one complex molecule and carbon atom C23′ of phenyl group of the next complex molecule with the distance C8…C23′ of 3.36(1) Å.

**Figure 8:**
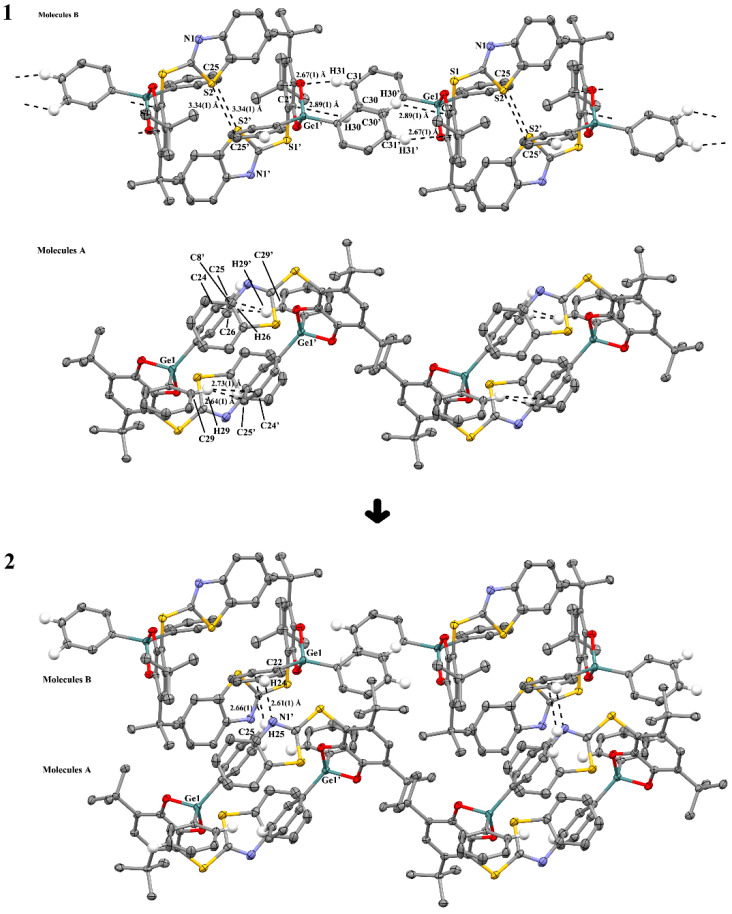
(**1**) The intermolecular interactions between molecules A (bottom) and B (top); (**2**) the intermolecular interactions between molecules A and B in crystal of **8**.

The crystal cell of **8** contains two types of molecules (independent molecules A and B), which demonstrate different patterns of intermolecular interactions (Figure 8).

Molecule B forms pairs via T-type contacts: the contact C31–H31…π-system C(1′-6′) is 2.67(1) Å (the corresponding distances C31–H31…C1′-C6′ vary in the range of 2.74–3.37(1) Å). The intermolecular interactions C30–H30…C2′ (2.89(1) Å) and S2…C25′ (3.34(1) Å) between these pairs form a linear motif (Figure 8(1), top). Molecule A also forms dimers by the intermolecular interaction between phenyl groups with the short contacts C29–H29…C24′ of 2.73(1) Å and C29–H29…C25′ of 2.64(1) Å (Figure 8(1), bottom), as well as due to the interaction between phenyl group and carbon C8′ of benzothiazole group, the distance C26–H26…C8′ is 2.80(1) Å. Moreover, the interaction between π-systems of phenyl rings C(22–27) takes place in this dimer, and the corresponding distance π-system C(22–27)… π-system C(22′–27′) is 3.67(1) Å. At the same time, in contrast to molecule B, there are no specific interactions between these dimers.

Further, molecules A and B are connected by the short contacts of T-type, namely C25A–H25A…π-system C(22B–27B) of 2.66(1) Å (the range of the distances C25A–H25A…C22B-C27B is 2.87–3.15(1) Å) and by the interactions between nitrogen atoms N1 of benzothiazole fragment and phenyl group (the distance N1A…H24B–C24B is 2.61(1) Å) (Figure 8(2)).

### 2.3. Electrochemistry

**Figure 9:**
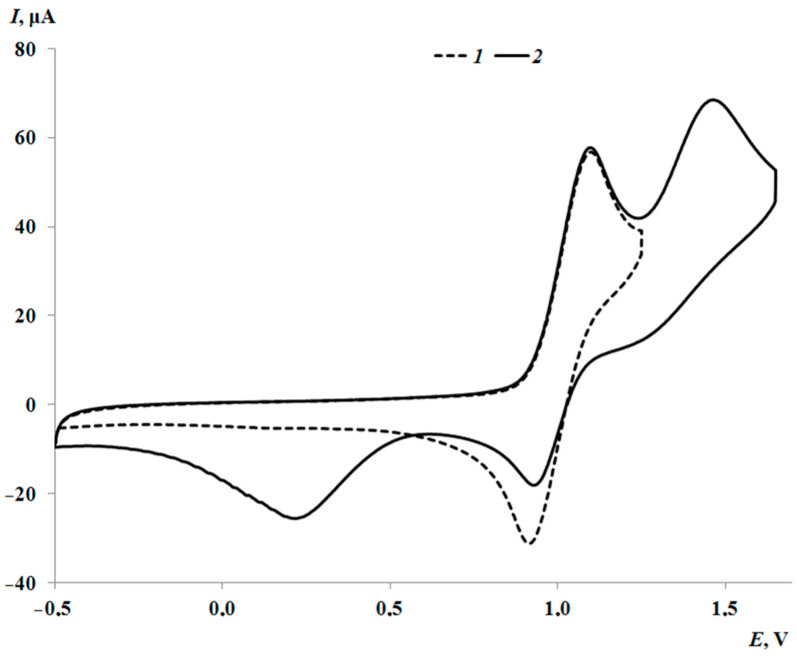
The CV curves of **1** at the potential ranges from −0.5 to 1.25 V (curve 1); from −0.5 to 1.65 V (curve 2) (CH_2_Cl_2_, GC anode, Ag/AgCl/KCl(sat.), 0.15 M nBu_4_NClO_4_, C = 3·10^−3^ M, argon).

**Figure 10:**
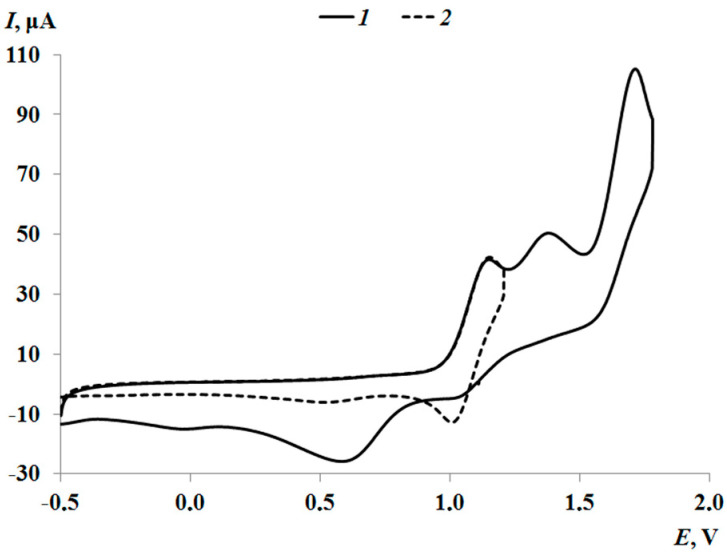
The CV curves of **5** at the potential ranges from −0.5 to 1.21 V (curve 1); from −0.5 to 1.78 V (curve 2) (CH_2_Cl_2_, GC anode, Ag/AgCl/KCl(sat.), 0.15 M nBu_4_NClO_4_, C = 3·10^−3^ M, argon).

It was previously shown that the coordination of metal ions or organometallic compounds with redox-active ligands in different oxidation states increases the number of available redox forms [76,77,78,79]. This is especially important for non-transition elements because bonding with such ligands makes it possible to expand their valence capabilities [80,81,82,83]. Electrochemical methods, including CV, are convenient for studying redox transformations of complexes with redox-active ligands. The electrochemical data on organogermanium(IV) catecholates are given in Table 2 in comparison with related triphenylantimony(V) catecholates [84,85,86]. The target complexes undergo oxidation in two or three successive stages in the potential range from 0.5 to 2.0 V (Figure 9 and Figure 10).

The previously studied six- and four-coordinated germanium(IV) catecholate complexes are characterized by one irreversible oxidation stage in the potential range from 0.80 to 0.93 V (vs. Ag/AgCl), both in aqueous solutions and in aprotic solvents [24,87,88]. A feature of sterically hindered catecholate ligands is the possibility of stabilizing the one-electron oxidized o-semiquinone form in the coordination sphere of the metal.

**Figure 11:**
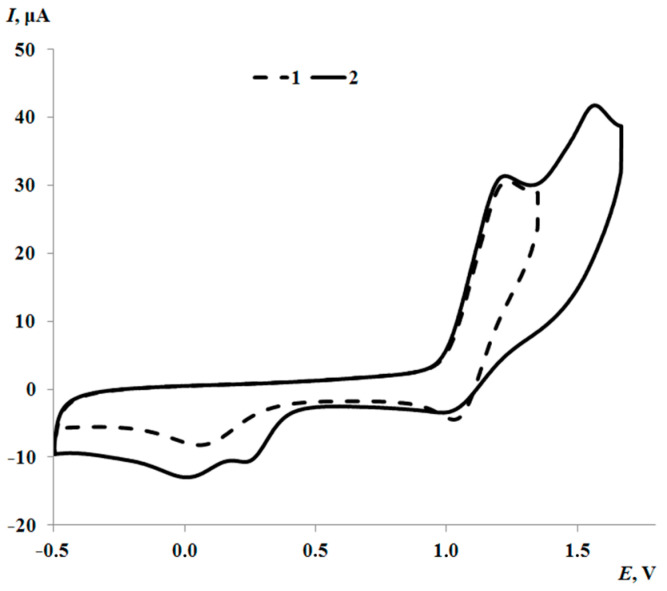
The CV curves of **2** at the potential ranges from −0.5 to 1.34 V (curve 1); from −0.5 to 1.67 V (curve 2) (CH_2_Cl_2_, GC anode, Ag/AgCl/KCl (sat.), 0.15 M nBu_4_NClO_4_, C = 1.5·10^−3^ M, argon).

**Scheme 2:**
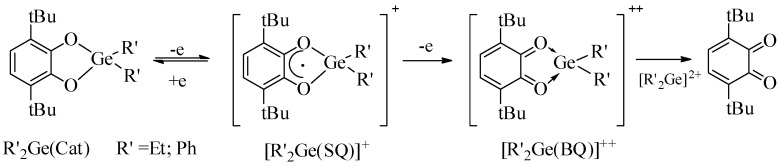
Electrochemical oxidation of **1** and **2**.

Compounds **1**, **2**, and **10** without an additional redox center such as a thioether group undergo oxidation in two anodic stages (Figure 11). The first oxidation is the quasi-reversible one-electronic stage, which leads to the generation of a monocationic complex ([R’_2_Ge(SQ)]^+^) (Scheme 2). The second peak corresponds to the further ligand oxidation with the formation of unstable dicationic derivatives ([R’_2_Ge(BQ)]^++^). This intermediate contains the ligand in the *o*-quinoid form. The elimination of free o-benzoquinone accompanies the decomposition of this dication complex.

Based on the current ratios (I_c/Ia_) (Table 2), the monocationic complex [Et_2_Ge(SQ)]^+^ generated under the CV conditions is more stable as compared to the oxidized forms of compounds **2** and **10** (Figure S19). Changing the position of the *tert*-butyl groups in the catecholate ring has no significant effect on the E^ox1^_1/2_ values for **2** and **10**. The substitution of the phenyl groups at the germanium atom in **2** with ethyl groups in **1** leads to the oxidation potential shift by 0.1 V. In the case of these complexes, the second anodic peak characterizing the formation of a dication derivative is fixed at close potentials. At the same time, a shift of E^ox2^_p_ to the cathodic region is observed for **10**. This indicates that the boundary redox orbitals in the generated monocation are approaching each other. The decrease in the current ratio value for **2** and **10** is in good agreement with the appearance of a peak on the reverse scan of the CV curve. This cathodic peak corresponds to the reduction in the product forming after electron transfer. For **1**, the peak at 0.18 V (Figure 9) appears only as a result of expanding the potential sweep range to 1.6 V.

To confirm the proposed scheme of redox transformations leading to the decoordination of *o*-benzoquinone, microelectrolysis was carried out at a potential of 1.25 V (1.5 h) using complex **1** as the example. The electrolysis is accompanied by the solution coloration and the appearance of a quasi-reversible peak at −0.52 V in the cathodic region (Figure S20). This peak is typical of the 3,6-di-*tert*-butyl-*o*-benzoquinone reduction. The amount of electricity consumed corresponds to a transfer of 1.7 electrons. The generated monocationic complex is unstable under electrolysis conditions and decomposes with the formation of an *o*-semiquinone radical anion. The disproportionation of this intermediate causes the *o*-benzoquinone formation.

The effect of the solvent nature on the electrochemical properties was studied for **1** and **2**. It was found that the use of coordinating acetonitrile instead of dichloromethane contributes to a 1.5-fold increase in the first peak current intensity as well as to its shift to the cathodic region by 0.07 and 0.10 V for **1** and **2**, respectively (Figures S21 and S22). The current ratio reduces to 0.6 in the case of **1**, while an irreversible peak is observed for **2**. The number of transferred electrons reaches **2**. In both cases, the second anodic peak is ill-defined. These data are in good agreement with the previously observed change in the electrochemical picture for triphenylantimony(V) catecholate complexes upon coordination of nitrogen-containing compounds [89,90]. The coordination of acetonitrile at the germanium atom contributes to the destabilization of the complex oxidized form and a change in the electro-oxidation mechanism from one to two electrons.

Depending on the electrochemical behavior, diphenylgermanium(IV) catecholate complexes **3**–**9** with thioether linker in the ligand can be divided into two groups: the first one consists of **3**–**5** and **7**, and the second group includes **6**, **8**, and **9**. This division is determined depending on the demonstrated electrochemical activity. The first group of compounds is characterized by the presence of two anodic peaks on the CV curves (Figure 10) at the potential range from 1.0 to 1.60 V. The first oxidation peak is quasi-reversible (Figures S23 and S24). In the case of **3**, **4**, and **7**, the presence of a thioether group in the catecholate ring causes a slight drift of E^ox1^_1/2_ to the anodic region (Table 2) as compared to **10**.

**Figure 12:**
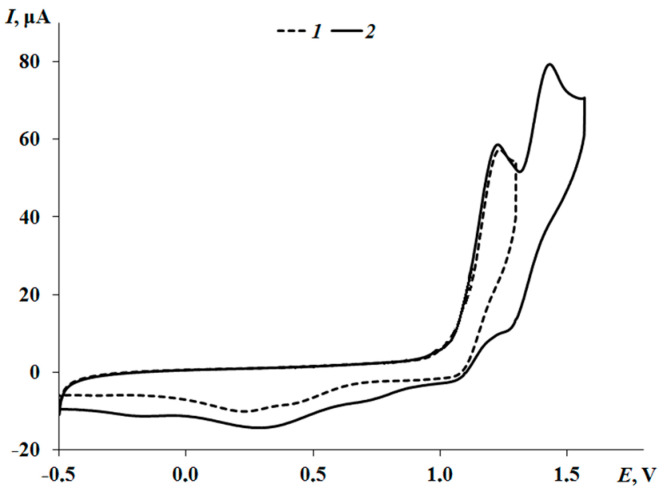
The CV curves of **7** at the potential ranges from −0.5 to 1.30 V (curve 1); from −0.5 to 1.57 V (curve 2) (CH_2_Cl_2_, GC anode, Ag/AgCl/KCl(sat.), 0.15 M nBu_4_NClO_4_, C = 3·10^−3^ M, argon).

Such behavior indicates the electron-withdrawing effect of the thioether bridge fragment. It is consistent with previous results for triphenylantimony complexes [85]. On the contrary, there is a shift in the oxidation potential to the cathodic region for **5**. Such an electrochemical picture can be explained by the coordination of the hydroxyl group to the germanium atom, which will increase the electron density and facilitate the oxidation process. Based on the current ratio values (Table 2), in the series of electrogenerated monocationic complexes, the more stable is cation [Ph_2_Ge(4,6-^s^SQ-BuOH)]^+^, and the less stable is [Ph_2_Ge(4,6-^s^SQ-Ver)]^+^. The second anodic peaks for compounds **3**–**5** are irreversible and characterize the formation of unstable dicationic derivatives, which decompose with an *o*-benzoquinone release. Microelectrolysis of **4** at a controlled potential of 1.3 V (1 h) is accompanied by a dark cherry coloration of the solution and the appearance of a cathodic peak at −0.45 V, which corresponds to the reduction in electrogenerated *o*-benzoquinone [91]. The more intense third anodic peak is observed on the CV curves in the potential range from 1.70 to 1.86 V. This peak can be attributed to the oxidation of thioether moiety in the ligands. A feature of complex **7** with the veratrol group in the ligand is the second quasi-reversible peak fixed on the CV curve (Figure 12) at E^ox2^_1/2_ = 1.38 V (I_c_/I_a_ = 0.7).

**Scheme 3:**
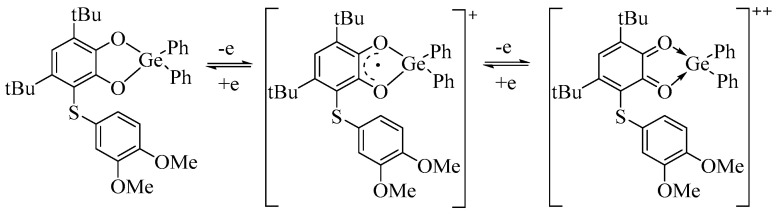
Electrochemical oxidation of **7**.

The presence of a redox-active veratrol group contributes to the stabilization of the dicationic derivative of **7**. Therefore, the redox transformation scheme of this complex can be represented as follows (Scheme 3).

The peak observed at higher anodic potentials (1.72 V) is related to the further oxidation of the dicationic derivative involving the thioether group in the ligand (Figure S25). An increase in the potential sweep range to 1.8 V leads to the disappearing reversibility of the two oxidation peaks, which points out the destruction of the complex.

**Figure 13:**
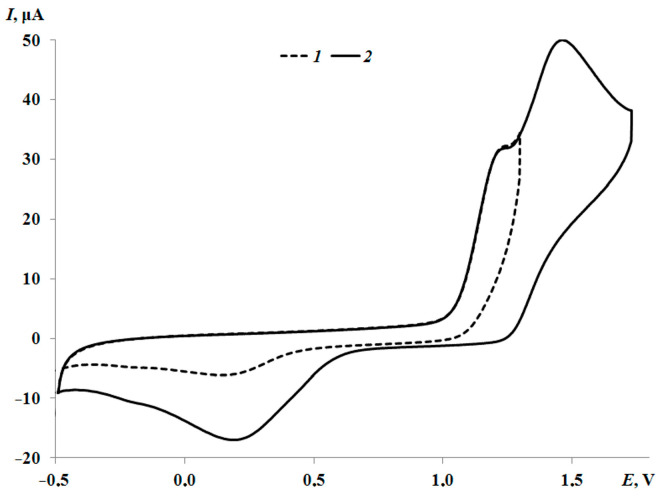
The CV curves of **8** at the potential ranges from −0.5 to 1.30 V (curve 1); from −0.5 to 1.72 V (curve 2) (CH_2_Cl_2_, GC anode, Ag/AgCl/KCl(sat.), 0.15 M nBu_4_NClO_4_, C = 2·10^−3^ M, argon).

The difference between the compounds of the second group and compounds **3**–**5** and **7** lies in the irreversible nature of the first oxidation stage (Figure 13 and Figures S26 and S27). Monocationic complexes formed during the electro-oxidation of compounds **6**, **8**, and **9** are unstable and undergo destruction. Complexes with heterocyclic fragments in ligands show a shift of the oxidation peak potentials towards more positive values compared to substances of the first group (Table 2). The second anodic peak of complexes **6** and **8** is observed in the same potential range as in the case of the SQ/BQ transition for other complexes. The presence of only two oxidation peaks in the CV curves is noted for complex **9** with a fragment of sterically hindered phenol (Figure S27). The second oxidation peak is observed at 1.51 V, which is close to the free ligand oxidation potential (1.49 V) [86].

A comparative analysis of the E^ox1^_1/2_ values of germanium(IV) catecholates and previously studied triphenylantimony(V) complexes with similar ligands (Table 2) showed that the oxidation potentials of fourcoordinate germanium compounds are shifted to the anodic region by 0.18–0.24 V. The oxidation potential of the diphenyltin(IV) complex with catecholate ligand was also observed at a potential significantly shifted to the cathodic region [78]. The obtained results for organogermanium(IV) catecholate suggest a more rigid character of the [R’_2_Ge]^2+^ moiety as a Lewis acid.

### 2.4. Radical Scavenging and Antioxidant Activity

Previously, the antioxidant activity of Ge-132^47^, germanium(IV) catecholate complexes [87,88], as well as organogermanium derivatives with ascorbic acid fragments and polyphenolic ligands [42,43,44] was discovered against DPPH and hydroxyl radicals. The antiradical activity of complexes **1**–**10** was studied in the reactions with a stable DPPH radical, the ABTS radical cation (Table 3).

In the series of organogermanium complexes, the IC_50_ and TEC_50_ indicators vary over a wide range of values. In the case of **1**, **2**, and **10**, the antiradical activity is influenced by both the substituents on the germanium(IV) atom and the position of *tert*-butyl groups in the catecholate ring. The ethyl substitution by a phenyl group for complexes **1** and **2** leads to a decrease in IC_50_ to 59 µM with a comparable time to reach the equilibrium state (TEC_50_). There is a two-fold lowering in neutralizing activity towards the DPPH radical for **10** compared to **2**. The appearance of a thioether group in the catecholate ring contributes to a decrease in the antiradical properties in the series of complexes **3**, **5**–**7**, and **4**, with the exception of compounds **8** and **9**.

Complexes **4** and **7** are characterized by increased IC_50_ and TEC_50_ values, and they have a weak activity against the DPPH radical. The best indexes in the DPPH assay were obtained for complexes **9** and **8** containing fragments of sterically hindered phenol or benzothiazole. The number of neutralized DPPH molecules (n_DPPH_) reaches two in the case of **8**, and this value is three molecules for **9**. Generally, a value n_DPPH_ for the catecholate ligand can achieve two units as this fragment is a two-electron donor. In the case of **9**, the increase in n_DPPH_ to more than two is due to the participation of the phenolic group in the reaction with DPPH. In terms of the IC_50_ parameter, the activity of complexes **8** and **9** is close to or even exceeds the data for Trolox. Using the example of complexes **2** and Ph_3_Sb(3,6-Cat) with a similar ligand, it was shown that replacing one organometallic fragment with another leads to reduced neutralizing activity of complex **2** towards DPPH at comparable TEC_50_ values. A similar effect is observed for compound **3** and Ph_3_Sb(4,6-^s^Cat-Bu). However, the TEC_50_ parameter for organogermanium complex is significantly reduced in comparison with Ph_3_Sb(4,6-^s^Cat-Bu).

Upon interaction with the ABTS radical cation, the minimum IC_50_ values (Table 3) were obtained for compounds **5** and **7**–**9**. A feature of these substances is the presence in the catecholate ligand of electron-rich fragments at the sulfur atom such as benzothiazole, veratrole, and sterically hindered phenol, as well as a butanol residue. These results for compounds **8** and **9** are in good agreement with the minimum IC_50_ parameters in the DPPH test. It indicates the high neutralizing activity of these complexes towards ABTS^∙+^ and diphenylpicrylhydrazyl radical. Based on the ABTS_TEAC_ values, all studied complexes are significantly inferior in the radical scavenging activity of Trolox.

**Figure 14:**
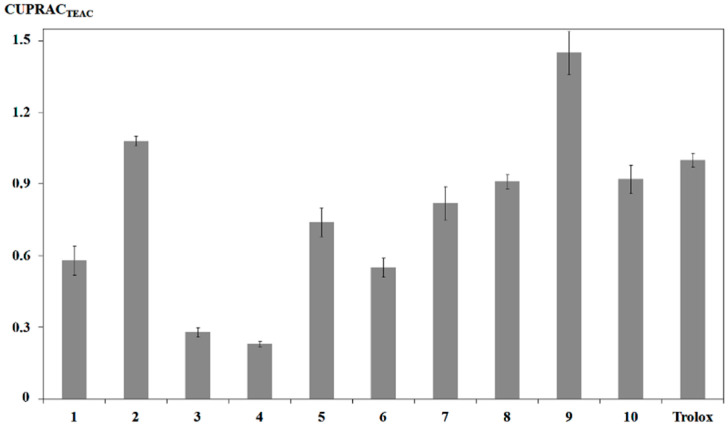
The Cu^2+^ reducing activity of **1**–**10** in Trolox equivalents (CUPRAC_TEAC_).

To assess the antioxidant capacity of organogermanium(IV) complexes **1**–**10** in Trolox equivalents, their activity towards Cu^2+^ ions in the presence of neocuproine (CUPRAC_TEAC_) was studied (Figure 14).

The minimum IC_50_ indexes in ABTS assay for complexes **2**, **5**, and **7**–**9** are confirmed by the CUPRAC_TEAC_ values (0.74–1.45). The activity of **2** and **10** in the CUPRAC test is comparable to Trolox. The increase in CUPRAC_TEAC_ is observed for **9**. The presence of an antioxidant phenolic group can explain this behavior. A slight promotion in antioxidant capacity is observed for **2** as compared to Ph_2_Ge(3,5-Cat) (Figure 14). A comparative analysis of the data for complexes **3**, **4**, and **10** showed that the introduction of an electron-withdrawing S-alkyl group into the catecholate ring causes the reduction in the radical scavenging and antioxidant properties in the ABTS and CUPRAC tests.

**Figure 15:**
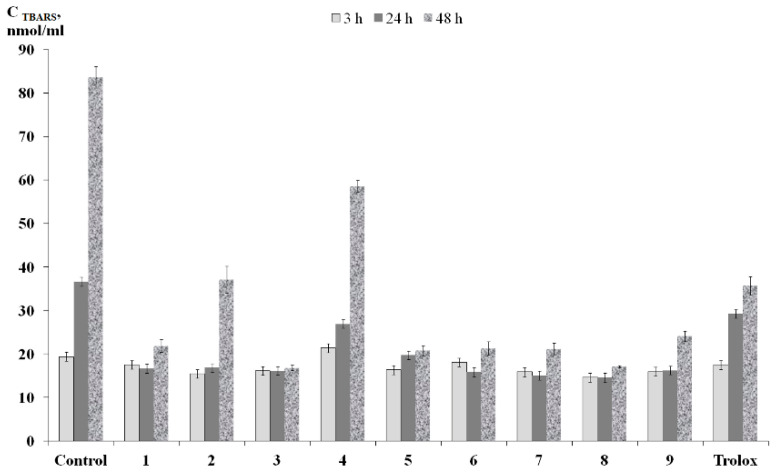
The concentration of TBARS in rat liver homogenates in vitro, in the presence of **1**–**10**, Trolox, and without additives (Control) during the incubation 3, 24, and 48 h (concentration of compounds in the reaction medium is 0.1 mM). The results are expressed as mean ± standard deviation (*p* < 0.001).

Earlier, it was shown that the organogermanium compounds can reduce lipid peroxidation, protect the cell membrane from injury, and reduce the lipid peroxide level in plasma, liver, or brain tissues [93]. Similar results were also obtained by our group when studying the antioxidant activity of triphenylantimony(V) catecholate complexes in vitro and in vivo [94,95,96,97]. It was found that the presence of a catecholate ligand makes it possible to neutralize the toxic effect of the organometallic fragment. The presence of various groups in the chelating catecholate ligands makes it possible to modulate the biological activity, including the anti-/pro-oxidant properties of coordination compounds of non-transition elements. It is interesting to estimate the influence of oranogermanium(IV) compounds **1**–**10** on the lipid peroxidation (LP) reaction of the rat (Wistar) liver homogenate as a non-enzymatic process induced by Fe(II) ions (in vitro). The lipid peroxidation of the rat liver homogenates was assessed by the accumulation of TBARS products. The samples of the homogenates were divided as follows: one control (blank experiment) and homogenates with additives of compounds **1**–**10** and Trolox. The content of TBARS was determined by measuring the absorbance of the solution at 535 nm using UV–Vis spectroscopy (Figure 15).

At the first stage (3 h) of the lipid peroxidation process, most complexes have an inhibitory effect. In the presence of target compounds, the TBARS concentration is decreased by 7–25% compared to the control sample. Compound **8** with a benzothiazole group has the greatest antioxidant effect as the intensity of lipid peroxidation decreases by 25%. At the same time, the additive of complex **4** is characterized by a weak promoting effect on LP. Incubation of the homogenate for 24 h leads to a systematic increase in the TBARS content in the control sample. In the presence of additives of organogermanium(IV) catecholates, a significant decrease in the amount of TBARS by 46–60% is observed. An inversion of properties from pro-oxidant to antioxidant is noted for compound **4**, but the inhibitory activity of this complex is not high. The maximum effect on the TBARS value is observed in the presence of compounds **6**–**8** with additional (hetero-)aromatic fragments in the structure of catecholate ligands. At this stage (24 h), germanium(IV) complexes have a more pronounced influence than the standard antioxidants such as Trolox.

After 48 h of incubation, a significant decrease in the concentration of TBARS by 72–80% happened in the samples with compounds **1**, **3**, and **5**–**9**. The antioxidant effect of this series of substances exceeds the results obtained for Trolox. Complexes **2** and **4** turned out to be less active inhibitors of LP. Compounds **1** and **2** initially have a similar effect on the lipid peroxidation process; however, the effectiveness of complex **2** with phenyl groups at the germanium atom is lowered with the elongation of experiment time. It is worth noting that the antioxidant action gradually increases for complexes **1**, **3**, and **5**–**9** during the experiment time. In the presence of **3**, **5**, **6**, and **8**, the concentration of TBARS varied slightly throughout the experiment indicating an induction period in the lipid peroxidation. These substances have a prolonged inhibitory action on the LP process.

## 3. Materials and Methods

### 3.1. General

The starting reagents were commercially available Et_2_GeCl_2_ (98%, Aldrich, Munich, Germany), Ph_2_GeCl_2_ (97%, Aldrich, Munich, Germany), 2,2′-azino-bis(3-ethylbenzothiazoline-6-sulfonic acid), ABTS^+^ (≥98%, TCI, Tokyo, Japan), 2,2-diphenyl-1-picrylhydrazyl, DPPH (98%, Aldrich, Getmany), thiobarbituric acid (≥98%, Merck, Darmstadt, Germany), neocuproine (2,9-dimethyl-1,10-phenanthroline) (99%, Aldrich, Germany), phosphate-buffered saline (PBS, Arlington, VA, USA) pH 7.4 (Sigma, Germany), 6-hydroxy-2,5,7,8-tetramethylchromane-2-carboxylic acid (Trolox) (97%, Aldrich, Germany), 3,5-di-*tert*-butylcatechol (98%, Aldrich, Germany), and trichloroacetic acid (≥99%, Sigma-Aldrich, Munich, Germany), which were used without further purification in the synthesis of the target compounds and biological tests. Catechol thioethers were synthesized by known methods [86,91,98,99]. Solvents were purified following standard procedures [100].

### 3.2. Spectroscopic Studies

The IR spectra were recorded on an FSM-1201 FT-IR spectrometer (LLC “Monitoring”, Saint Petersburg, Russia) in KBr pellets. The NMR spectra were measured in CDCl_3_ on Bruker Avance HD 400 or Bruker Avance DPX 300 spectrometers (Bruker Biospin AG, Faellanden, Switzerland) with a frequency of 400 or 300 MHz (^1^H) and 100 or 75 MHz (^13^C{^1^H}), respectively, using Me_4_Si as an internal standard. The chemical shift values are given in ppm with reference to the solvent, and the coupling constants (J) are given in Hz. The elemental analysis was carried out on a Euro EA 3000 (C,H,N) elemental analyzer (EuroVector Srl, Redavalle, Italy). Mass spectra (HRMS) were recorded on a Bruker UHR-TOF Maxis™ Impact mass spectrometer (Bruker Daltonik GmbH, Bremen, Germany). The UV–Vis spectra were recorded with an SF-104 spectrophotometer (AKVILON, Podol’sk, Russia) in a range of 300–600 nm or a Multiskan Sky microplate spectrophotometer (Thermo Scientific, Waltham, MA, USA).

### 3.3. Single-Crystal X-ray Analysis

The X-ray diffraction data were collected on Bruker D8 Venture (for **1**, **2**, **6**, and **8**) and Bruker SMART APEX II (for **3**) diffractometers equipped with a CCD detector and a monochromatic radiation source (Mo-Kα, λ = 0.71073 Å). Semi-empirical absorption correction was applied by the SADABS program [101]. The structures were solved by direct methods and refined by the full-matrix least squares in the anisotropic approximation for non-hydrogen atoms. Hydrogen atoms of the carbon-containing ligands were geometrically generated and refined in the riding model. The calculations were carried out by the SHELX-2014 program package [102] using Olex2 [103]. Crystallographic data for structures reported in this paper were deposited with the Cambridge Crystallographic Data Center: CCDC numbers 2345920 (**1**), 2346948 (**2**), 2345921 (**3**), 2346949 (**6**), and 2347014 (**8**). The crystallographic parameters and X-ray diffraction experimental parameters are given in Table S1.

### 3.4. Synthesis and Characterization

Synthesis of organogermanium(IV) catecholates R_2_Ge(Cat) was carried out as follows: the equivalent amounts of R_2_GeCl_2_ (0.3 mmol) dissolved in toluene (2 mL) were added to a toluene solution of ligand (0.3 mmol, toluene 5 mL) under argon. Further, 2 equiv. of triethylamine was added to the solution under extensive stirring. After the addition of reagents was complete, the reaction mixture was stirred for 4–5 h. Then, the reaction mixture was stored at 5 °C for 24 h. The white precipitate of triethylammonium hydrochloride was filtered off and washed with n-hexane (15 mL). The toluene in the filtrate was removed under reduced pressure, and the residue was diluted in n-hexane. Both hexane portions were combined and filtered again to remove the rest of the triethylammonium hydrochloride. The filtrate was concentrated under a reduced pressure to a half volume and stored at −18 °C for 5–7 days. The X-ray-suitable crystals of compounds **1**–**3**, **6**, and **8** were collected by decantation and dried under reduced pressure.

Et_2_Ge(3,6-Cat) (**1**): The yield of **1** as colorless crystals was 52% (0.055 g). IR (KBr, ν, cm^−1^): 3082, 2967 2951, 2907, 2876, 1655, 1558, 1490, 1457, 1399, 1384, 1362, 1308, 1284, 1261, 1228, 1203, 1145, and 1027; ^1^H NMR (300 MHz, CDCl_3_, ppm): 1.16 (t, ^3^J(H,H) = 7.8 Hz, 6H, CH_3_CH_2_), 1.40 (s, 18H, tBu), 1.37–1.47 (m, 4H, CH_3_CH_2_), and 6.66 (s, 2H, arom. C_6_H_2_); ^13^C{^1^H} NMR (75 MHz, CDCl_3_, ppm): 6.47, 12.11, 29.22, 34.19, 115.58, 133.43, and 149.16; Calcd. for C_18_H_30_GeO_2_ (%): C, 61.58; H, 8.61; and found (%): C, 61.67; H, 8.74.

Ph_2_Ge(3,6-Cat) (**2**): The yield of **2** as colorless crystals was 61% (0.082 g). IR (KBr, ν, cm^−1^): 3075, 3054, 3030, 2979, 2952, 2908, 2866, 1595, 1485, 1464, 1435, 1397, 1387, 1358, 1308, 1285, 1264, 1233, 1207, 1184, 1145, 1049, and 1026; ^1^H NMR (300 MHz, CDCl_3_, ppm): 1.46 (s, 18H, tBu), 6.77 (s, 2H, arom. C_6_H_2_), 7.49–7.54 (m, 4H, Ph), 7.56–7.62 (m, 2H, Ph), and 7.73–7.78 (m, 4H, Ph); ^13^C{^1^H} NMR (75 MHz, CDCl_3_, ppm): 29.41, 34.32, 116.24, 129.00, 130.85, 132.09, 133.93, 134.10, and 148.54; Calcd. for C_26_H_30_GeO_2_ (%): C, 69.84; H, 6.76; and found (%): C, 69.73; H, 6.90.

Ph_2_Ge(4,6-^s^Cat-tBu) (**3**): The yield of **3** as colorless powder was 46% (0.074 g). IR (KBr, ν, cm^−1^): 3072, 3055, 2984, 2954, 2910, 2866, 1584, 1543, 1485, 1458, 1429, 1394, 1373, 1361, 1310, 1253, 1234, 1183, 1150, 1102, 1094, and 1024; ^1^H NMR (300 MHz, CDCl_3_, ppm): 1.31 (s, 9H, tBu), 1.47 (s, 9H, tBu), 1.54 (s, 9H, tBu), 6.91 (s, 1H, arom. C_6_H_1_), 7.46–7.61 (m, 6H, Ph), and 7.72–7.77 (m, 4H, Ph); ^13^C{^1^H} NMR (75 MHz, CDCl_3_, ppm): 29.41, 32.19, 34.93, 37.15, 47.49, 115.48, 116.70, 129.01, 130.58, 132.16, 134.12, 134.34, 144.17, 145.70, and 153.41; Calcd. for C_36_H_44_GeO_2_S (%): C, 70.49; H, 7.23; and found (%): C, 70.57; H, 7.41.

Ph_2_Ge(4,6-^s^Cat-Ad) (**4**): The yield of **4** as colorless powder was 49% (0.090 g). IR (KBr, ν, cm^−1^): 3072, 3055, 2954, 2903, 2846, 1584, 1485, 1455, 1433, 1391, 1371, 1356, 1342, 1310, 1295, 1252, 1235, 1185, 1150, 1093, 1049, and 1036; ^1^H NMR (300 MHz, CDCl_3_, ppm): 1.30 (br.d.m, ^2^J(H,H) = 12.0 Hz, 3H, Ad), 1.45 (br.d.m, ^2^J (H, H) = 12.0 Hz, 3H, Ad), 1.48 (s, 9H, tBu), 1.55 (s, 9H, tBu), 1.77 (br.s, 3H, Ad), 1.91 (br.s, 6H, Ad), 6.91 (s, 1H, arom. C_6_H_1_), 7.45–7.62 (m, 6H, arom. C_6_H_5_), and 7.73–7.80 (m, 4H, arom. C_6_H_5_); ^13^C{^1^H} NMR (75 MHz, CDCl_3_, ppm): 29.41, 30.55, 32.34, 34.94, 36.13, 37.14, 44.17, 50.13, 115.17, 115.44, 129.01, 130.61, 130.67, 132.19, 134.16, 144.36, 145.68, and 153.52; Calcd. for C_36_H_44_GeO_2_S (%): C, 70.49; H, 7.23; and found (%): C, 70.57; H, 7.41.

Ph_2_Ge(4,6-^s^Cat-BuOH) (**5**): The yield of **5** as colorless powder was 42% (0.070 g). IR (KBr, ν, cm^−1^): 3519, 3072, 3052, 2954, 2910, 2869, 1584, 1483, 1458, 1432, 1392, 1377, 1311, 1254, 1234, 1200, 1153, 1098, 1090, 1048, and 1033; ^1^H NMR (400 MHz, CDCl_3_, ppm): 1.45 (s, 9H, tBu), 1.54 (s, 9H, tBu), 1.55–1.70 (m, 4H, CH_2_ of nBu), 2.95 (t, ^3^J(H,H) = 7.5 Hz, 2H, SCH_2_), 3.54 (t, ^3^J(H,H) = 6.3 Hz, 2H, OCH_2_), 6.89 (s, 1H, arom. C_6_H_1_), 7.45–7.55 (t.m, ^3^J(H,H) = 7.4 Hz, 5H: 4H, arom. C_6_H_5_ + 1H, OH), 7.55–7.61 (m, 2H, arom. C_6_H_5_), and 7.74 (d.m, ^3^J(H,H) = 7.4 Hz, 4H, arom. C_6_H_5_); ^13^C{^1^H} NMR (100 MHz, CDCl_3_, ppm): 25.57, 29.36, 31.47, 32.25, 34.80, 34.89, 36.91, 62.49, 115.27, 117.07, 129.04, 130.46, 132.26, 134.11, 134.13, 142.68, 145.91, and 152.46; HR-MS: found *m*/*z*: 575.1626 [M + Na]^+^; C_30_H_38_GeNaO_3_S; and Calcd. *m*/*z*: 575.1651.

Ph_2_Ge(4,6-^s^Cat-Fur) (**6**): The yield of **6** as colorless powder was 65% (0.110 g). IR (KBr, ν, cm^−1^): 3147, 3116, 2984, 2961, 2910, 2870, 1584, 1503, 1482, 1458, 1433, 1394, 1385, 1311, 1256, 1235, 1173, 1147, 1125, 1101, 1090, and 1070; ^1^H NMR (400 MHz, CDCl_3_, ppm): 1.44 (s, 9H, tBu), 1.46 (s, 9H, tBu), 4.18 (s, 2H, SCH_2_), 5.86 (d, ^3^J(H,H) = 3.1 Hz, 1H, arom. C_4_H_3_O), 6.17 (dd, ^3^J(H,H) = 3.1 and 1.9 Hz, 1H, arom. C_4_H_3_O), 6.86 (s, 1H, arom. C_6_H_1_), 7.48–7.54 (m, 1H, arom. C_4_H_3_O), 7.48–7.54 (t.m, ^3^J(H,H = 7.7 Hz, 4H, arom. C_6_H_5_), 7.56–7.62 (m, 2H, arom. C_6_H_5_), and 7.77 (d.m, ^3^J(H,H) = 8.1 Hz, 4H, arom. C_6_H_5_); ^13^C{^1^H} NMR (100 MHz, CDCl_3_, ppm): 29.35, 30.99, 31.26, 34.92, 36.78, 107.37, 110.33, 115.27, 116.09, 129.10, 130.38, 132.31, 134.14, 134.46, 141.60, 143.04, 145.92, 151.65, and 152.36; HR-MS: found *m*/*z*: 583.1320 [M + Na]^+^; C_31_H_34_GeNaO_3_S; and Calcd. *m*/*z*: 583.1338.

Ph_2_Ge(4,6-^s^Cat-Ver) (**7**): The yield of **7** as gray powder was 40% (0.074 g). IR (KBr, ν, cm^−1^): 3076, 3048, 2964, 2909, 2871, 2828, 1584, 1505, 1484, 1460, 1432, 1395, 1380, 1360, 1313, 1255, 1231, 1184, 1173, 1159, 1135, 1098, and 1031; ^1^H NMR (300 MHz, CDCl_3_, ppm): 1.47 (s, 9H, tBu), 1.55 (s, 9H, tBu), 3.62 (s, 3H, OCH_3_), 3.83 (s, 3H, OCH_3_), 6.51–6.68 (m, 3H, arom. C_6_H_3_), 6.99 (s, 1H, arom. C_6_H_1_), and 7.28–7.62 (m, 10H, arom. C_6_H_5_); ^13^C{^1^H} NMR (75 MHz, CDCl_3_, ppm): 29.32, 31.50, 35.01, 36.85, 55.53, 55.87, 109.90, 111.57, 115.36, 118.26, 128.15, 128.82, 130.10, 130.28, 132.16, 133.56, 134.13, 135.67, 143.87, 146.36, 148.96, and 152.71; Calcd. for C_34_H_38_GeO_4_S (%): C, 66.36; H, 6.22; and found (%): C, 66.21; H, 6.37.

Ph_2_Ge(4,6-^s^Cat-Het) (**8**): The yield of **8** as white powder was 53% (0.098 g). IR (KBr, ν, cm^−1^): 3055, 2986, 2955, 2910, 2867, 1585, 1485, 1456, 1426, 1396, 1382, 1360, 1308, 1254, 1235, 1157, 1124, 1101, 1092, and 1021; ^1^H NMR (400 MHz, CDCl_3_, ppm): 1.50 (s, 9H, tBu), 1.54 (s, 9H, tBu), 7.02 (s, 1H, arom. C_6_H_1_), 7.22 (t.m, ^3^J(H, H) = 7.6 Hz, 1H, arom. C_6_H_4_), 7.26–7.33 (t.m, ^3^J(H, H) = 7.6 Hz, 4H, arom. C_6_H_5_), 7.37 (t.m, J(H,H) = 7.7 Hz, 1H, arom. C_6_H_4_), 7.45–7.54 (m, 7H: 6H, arom. C_6_H_5_ + 1H, arom. C_6_H_4_), and 7.84 (d.m, ^3^J(H,H) = 8.1 Hz, 1H, arom. C_6_H_4_); ^13^C{^1^H} NMR (100 MHz, CDCl_3_, ppm): 29.27, 31.38, 35.14, 36.83, 111.88, 115.84, 120.55, 121.58, 123.52, 125.66, 128.84, 129.92, 132.20, 134.07, 135.45, 137.17, 143.32, 146.80, 153.32, 154.03, and 172.14; HR-MS: found *m*/*z*: 636.1043 [M + Na]^+^; C_33_H_33_GeNNaO_2_S_2_; and Calcd. *m*/*z*: 636.1062.

Ph_2_Ge(4,6-Cat-PhOH) (**9**): The yield of **9** as white powder was 44% (0.092 g). IR (KBr, ν, cm^−1^): 3637, 3076, 3052, 2957, 2910, 2873, 2832, 1573, 1507, 1475, 1464, 1448, 1432, 1394, 1432, 1394, 1377, 1360, 1307, 1271, 1255, 1240, 1182, 1156, 1134, 1100, 1092, 1066, and 1024; ^1^H NMR (400 MHz, CDCl_3_, ppm): 1.36 (s, 18H, tBu), 1.42 (s, 9H, tBu), 1.43 (s, 9H, tBu), 4.49 (s, 2H, SCH_2_), 5.16 (s, 1H, OH), 6.83 (s, 1H, arom. C_6_H_1_), 7.41 (s, 2H, arom. C_6_H_2_), 7.47–7.53 (t.m, ^3^J(H,H) = 7.3 Hz, 4H, arom. C_6_H_5_), 7.54–7.60 (m, 2H, arom. C_6_H_5_), and 7.82 (d.m, ^3^J(H,H) = 8.0 Hz, 4H, arom. C_6_H_5_); ^13^C{^1^H} NMR (100 MHz, CDCl_3_, ppm): 29.47, 30.18, 32.42, 34.34, 34.81, 35.85, 36.33, 114.91, 120.09, 127.38, 128.96, 129.56, 130.66, 132.10, 133.17, 134.38, 136.23, 138.57, 145.81, 150.40, and 153.16; HR-MS: found *m*/*z*: 721.2740 [M + Na]^+^; C_41_H_52_GeNaO_3_S; and Calcd. *m*/*z*: 721.2750.

### 3.5. Electrochemistry

Electrochemical studies were carried out using VersaSTAT-3 potentiostate (PAR) in three-electrode mode. The stationary glassy carbon (d = 2 mm) disk was used as the working electrode; the auxiliary electrode was the platinum-flag electrode. The reference electrode was Ag/AgCl/KCl (sat.) with the watertight diaphragm. All measurements were carried out under argon. The samples were dissolved in the pre-deaerated solvent. The scan rate (*v*) was 200 mV∙s^−1^. The supporting electrolyte 0.15 M Bu_4_NClO_4_ (99%, electrochemical grade, Fluka) was dried in a vacuum (48 h) at 50 °C. The concentration of compounds was 1–3 mmol. Compounds **1**–**10** showed a linear dependency of the first anodic current peak with the square root of the scan rate (*v*^1/2^), indicating a diffusion-controlled system.

Microelectrolysis of compounds **1** or **4** was performed at 25 °C under anaerobic condition (argon) in an undivided three-electrode cell (2 mL) in CH_2_Cl_2_ with 0.15 M Bu_4_NClO_4_. Platinum-flag electrodes with a surface area of 0.7 cm^2^ were applied. The concentration of compounds was 3 mmol. The electrolysis at controlled potential was performed at 1.25 V (**1**) or 1.30 V (**4**). A platinum wire and the standard Ag/AgCl/KCl reference electrode with a watertight diaphragm were used.

### 3.6. DPPH Assay

DPPH radical scavenging activity was performed according to the method of Bondet et al. [104]. A solution of the radical DPPH in CH_2_Cl_2_ (C_0_ = 50 µM) was prepared daily and protected from light. The solution of germanium complex in CH_2_Cl_2_ (0.02 mL) was added to 2 mL of a 50 µM solution of DPPH in CH_2_Cl_2_. The decrease in absorbance was determined at 527 nm (ε_max_ = 1.67∙10^5^ M^−1^∙cm^−1^) every 5 min until the reaction reached a plateau at room temperature. The parameter IC_50_ was the concentration of an antioxidant necessary for decreasing the amount of DPPH radical by 50% of the initial value. To determine IC_50_, the plot of the residual concentration of the stable radical vs. molar ratio, expressed as the number of moles of the complex per 1 mole of the stable radical, was constructed. All experiments were performed in triplicate at room temperature.

### 3.7. ABTS Assay

The antiradical activity of the complexes in the reaction with the radical cation 2,2′-azino-bis (3-ethylbenzothiazoline-6-sulfonic acid) (ABTS^+^) was measured by a known method [105]. We recorded changes in the absorption intensity of ABTS^+^ (λ = 734 nm), generated by the action of K_2_S_2_O_8_, in the presence of various concentrations of organogermanium(IV) complexes (5–100 µmol/L). Stock solutions of test compounds and Trolox with a concentration of 1.0 mM were prepared in DMSO. The IC_50_ parameter was calculated as the minimum compound concentration required to reduce the ABTS^∙+^ content by 50% of the initial value. The dependence of absorption on concentration in the ABTS test was determined for the studied complexes. The absorbance of the blank (40 μL DMSO and 40 μL of radical cation) assay was set as 100% radical. Trolox Equivalent Antioxidant Capacity (TEAC) was measured by comparing the slopes of plots obtained for each complex compared to that of Trolox. All measurements were carried out at least three times.

### 3.8. CUPRAC Assay

The Cu(II) ion-reducing (CUPRAC) assay was carried out following a known method [106]. The organogermanium complexes or Trolox solution (DMSO) and ethanol (96%) were added to the initial mixture to make the final volume, 2 mL. The concentration of compounds in test tubes ranged from 10 to 50 μM. Absorbance was measured at 450 nm on a spectrophotometer Akvilon SF-104 (Russia) against a reagent blank 30 min later. Trolox was used as the standard antioxidant for calculating TEAC (Trolox Equivalent Antioxidant Capacity). TEAC coefficient for this assay was determined by relating the molar absorptivity, ε, of the test samples to that of Trolox as follows: TEAC = ε/ε_Trolox_ (ε_Trolox_ = 1.79·10^4^ L·mol^−1^·cm^−1^).

### 3.9. Lipid Peroxidation Assay

Samples of rat liver (Wistar) (1:10 *w/v*) were prepared immediately before use in a phosphate-buffered medium (pH 7.4) using a homogenizer. The intensity of rat liver homogenate’s lipid peroxidation (LP) was assessed by the accumulation of carbonyl products forming a colored complex with thiobarbituric acid (TBARS) according to a previously described method [107]. The level of lipid peroxidation was measured as a non-enzymatic process by the addition of ascorbic acid and (NH_4_)_2_Fe(SO_4_)_2_. The influence of compounds on lipid peroxidation of the rat liver homogenates was carried out at 37 °C in phosphate buffer (pH 7.4) in the presence or absence of test compounds or vehicle (DMSO). The concentration of compounds in the medium was 0.1 mM. The concentration of TBARS was determined after 3, 24, and 48 incubation hours at 37°C. All experiments were performed using three independent experiments. Preliminary experiments were performed in the absence of the compound’s interaction with thiobarbituric acid. The values are expressed as mean % ± SD.

The samples for the experiments “in vitro” were received from the biology department. The samples were stored in the freezer at −70 °C and used as needed. All manipulations in experiments “in vitro” were conducted according to the International Rules of GLP (Good Laboratory Practice).

## 4. Conclusions

As a result of the exchange reaction between R’_2_GeCl_2_ and functionalized catechols, a series of new organogermanium(IV) catecholates was obtained with a preparative yield of up to 65%. The crystal structures of compounds **1**–**3**, **6**, and **8** were determined by X-ray diffraction analysis. The complexes are four-coordinate compounds of germanium(IV) containing the dianionic form of the redox-active ligands. The electrochemical behavior of target complexes was studied by cyclic voltammetry. Most of the synthesized complexes are characterized by a quasi-reversible one-electron stage of electro-oxidation, unlike previously studied germanium(IV) catecholates, which are oxidized irreversibly. The presence of two anodic peaks on the CV curves was observed for complexes **1**, **2**, and **10**. These peaks correspond to the formation of mono- and dicationic complexes containing different redox forms of the ligand. The stable monocationic complexes generated under electrochemical conditions can potentially be used as electrocatalysts for organic compound oxidation. In the case of complex **7**, the formation of relatively stable mono- and dicationic forms is observed by the CV. This allows to expand the valence capabilities of complexes due to the participation of an additional electron-active fragment in redox transformations. Accordingly, compound **7** can also potentially be considered as a one- or two-electron oxidizer.

The presence of a thioether group for complexes **3**–**7** leads to the fixation of the third redox transition because of the participation of this moiety in electrochemical transformations. The stability of electrogenerated monocationic complexes is influenced by both hydrocarbon groups at the germanium atom and substituents in the catecholate ligand. The oxidation potentials of the complexes are significantly shifted to the anodic region compared to the catecholate complexes of antimony(V) and tin(IV), which indicate the more rigid nature of the [R’_2_Ge]^2+^ fragment as a Lewis acid. Unlike most complexes, a feature of the electrochemical behavior of compounds **6**, **8**, and **9** with (hetero-)aromatic fragments at the sulfur atom is irreversible electro-oxidation. This behavior is a limiting factor to the use of these compounds as potential electrocatalysts. To solve the problem of electrogenerated monocationic complex stability, additional modifications of the redox-active catecholate ligand structure or the organometallic part may be required.

Furthermore, the synthesized compounds can act as the effective radical scavengers or paramagnetic labels due to the participation of the coordinated catecholate ligand in the electron transfer reactions. The radical scavenging research in the reaction with the DPPH radical, ABTS radical cation, and the CUPRAC test made it possible to identify several compounds with high neutralizing ability. Complexes **8** and **9** are characterized by the best antiradical and antioxidant activity among the studied compounds. However, the electro-oxidation of these substances occurred irreversibly at the first stage and involved the catecholate fragment transformations. Presumably, additional electron-donating groups (benzothiazole, phenolic) can make a predominant contribution to their antiradical activity due to the implementation of other mechanisms of antioxidant action (HAT or ET). In the process of lipid peroxidation, all complexes (except compound **4** with an adamantyl substituent at the sulfur atom) play the role of effective LP inhibitors. It was shown that the antioxidant effect of complexes **1**, **3**, and **5**–**9** increased during the incubation time. For compounds **3**, **5**, **6**, and **8**, a period of LP induction was observed when the concentration of lipid peroxidation products remained virtually unchanged for 48 h.

Thus, the molecular structure of the synthesized compounds was established, the mechanism of their electro-oxidation was proposed, and the radical scavenging and antioxidant activity of the target complexes was determined. Modification of the catecholate ligand due to the presence of a thioether linker allows one to introduce the hydrophobic, redox, or biologically active groups, achieving a significant variation in the electrochemical properties and biological activity of organogermanium(IV) complexes. The combination of antioxidant groups and lipophilic organometallic fragments can lead to significant modulation of the biological activity of such compounds. Given the great potential of germanium(IV) compounds in medicinal chemistry, further studies of such compounds are promising.

## Figures and Tables

**Table 1:** The selected bond lengths (Å) and angles (°) in germanium catecholates **1**–**3**, **6**, and **8**.

Bond	1	2 *	3	6 **	8 *, ***
Ge1–O1	1.8130(8)	1.801(1)/1.800(1)	1.805(1)	1.800(1)	1.816(2)/1.812(2)
Ge1–O2	1.8099(9)	1.802(1)/1.804(1)	1.813(2)	1.812(1)	1.817(2)/1.815(2)
Ge1–C	Ge1–C15 1.928(1)	Ge1–C15 1.913(2)/1.922(2)	Ge1–C19 1.913(2)	Ge1–C20 1.912(2)	Ge1–C22 1.910(2)/1.914(3)
	Ge1–C17 1.930(1)	Ge1–C21 1.909(2)/1.924(2)	Ge1–C25 1.916(2)	Ge1–C26 1.918(2)	Ge1–C28 1.916(3)/1.915(2)
O1–C1	1.378(1)	1.376(2)/1.387(2)	1.387(2)	1.385(2)	1.382(2)/1.380(3)
O2–C2	1.379(1)	1.384(2)/1.388(2)	1.371(2)	1.374(2)	1.372(3)/1.365(3)
C1–C2	1.406(2)	1.407(3)/1.401(3)	1.406(2)	1.406(2)	1.401(4)/1.401(3)
C2–C3	1.399(2)	1.395(2)/1.397(2)	1.402(3)	1.394(3)	1.390(3)/1.397(3)
C3–C4	1.400(2)	1.397(2)/1.396(3)	1.424(2)	1.413(3)	1.424(3)/1.418(4)
C4–C5	1.392(2)	1.388(3)/1.389(3)	1.403(2)	1.400(2)	1.390(4)/1.399(3)
C5–C6	1.399(2)	1.396(2)/1.397(2)	1.410(3)	1.404(3)	1.405(3)/1.401(3)
C1–C6	1.401(2)	1.396(2)/1.394(2)	1.390(2)	1.384(3)	1.393(3)/1.389(3)
S1–C3	-	-	1.786(2)	1.778(2)	1.781(3)/1.784(2)
S1–C7	-	-	1.875(2)	1.827(2)	1.747(2)/1.748(2)

* For two independent molecules A and B; ** O3–C8 1.375(2); O3–C11 1.373(3); C8–C9 1.349(3); C9–C10 1.433(3); C10–C11 1.340(3) Å; *** S2–C7 1.756(3)/1.760(3); S2–C9 1.748(3)/1.686(3); N1–C7 1.296(4)/1.316(4); N1–C8 1.391(3)/1.407(6); and C8–C9 1.400(4)/1.390(4) Å.

**Table 2:** Redox potentials of complexes **1**–**10** obtained by the CV method (CH_2_Cl_2_, GC-electrode, 0.15 M nBu_4_ClO_4_, Ar, vs. Ag/AgCl/KCl).

№	Compound	E^ox1^_1/2_, V	I_c_/I_a_	E^ox2^_p_, V	E^ox3^_p_, V
**1**	Et_2_Ge(3,6-Cat)	1.03	1.0	1.46	-
**2**	Ph_2_Ge(3,6-Cat)	1.13	0.6	1.45	-
**3**	Ph_2_Ge(4,6-^s^Cat-tBu)	1.15	0.4	1.47	1.78
**4**	Ph_2_Ge(4,6-^s^Cat-Ad)	1.17	0.6	1.45	1.86
**5**	Ph_2_Ge(4,6-^s^Cat-BuOH)	1.08	0.8	1.38	1.70
**6**	Ph_2_Ge(4,6-^s^Cat-Fur)	1.23 *	-	1.43	1.82
**7**	Ph_2_Ge(4,6-^s^Cat-Ver)	1.18	0.5	1.42 **	1.72
**8**	Ph_2_Ge(4,6-^s^Cat-Het)	1.22 *	-	1.48	-
**9**	Ph_2_Ge(4,6-Cat-PhOH)	1.12 *	-	1.51	-
**10**	Ph_2_Ge(3,5-Cat)	1.14	0.6	1.36	-
	^a^ Ph_3_Sb(4,6-Cat-PhOH)	0.89	0.8	1.39	-
	^a^ Ph_3_Sb(4,6-^s^Cat-Bu)	0.94	0.7	1.40	1.69
	^a^ Ph_3_Sb(3,6-Cat)	0.89	0.8	1.40	-

* The values of potential for the irreversible peaks; ** the potential value for the quasi-reversible peak; and ^a^ data for triphenylantimony(V) catecholates are from refs. [84,85,86].

**Table 3:** The values of IC_50_ in DPPH test (CH_2_Cl_2_, 298 K), index of IC_50,_ and TEAC (Trolox equivalents) in ABTS^+^ assay of **1**–**10**.

N	Compound	IC_50_ (DPPH), µM	TEC_50_, Min	IC_50_ (ABTS^+^), µM	ABTS_TEAC_
**1**	Et_2_Ge(3,6-Cat)	80.2 ± 2.7	55	115.5 ± 3.2	0.20 ± 0.02
**2**	Ph_2_Ge(3,6-Cat)	59.0 ± 0.5	60	37.3 ± 0.8	0.39 ± 0.09
**3**	Ph_2_Ge(4,6-^s^Cat-tBu)	60.1 ± 0.9	37	113.0 ± 4.5	0.21 ± 0.01
**4**	Ph_2_Ge(4,6-^s^Cat-Ad)	178.0 ± 3.6	120	76.6 ± 1.8	0.33 ± 0.01
**5**	Ph_2_Ge(4,6-^s^Cat-BuOH)	76.4 ± 1.0	80	32.0 ± 1.5	0.54 ± 0.04
**6**	Ph_2_Ge(4,6-^s^Cat-Fur)	85.6 ± 3.2	110	60.3 ± 1.9	0.29 ± 0.03
**7**	Ph_2_Ge(4,6-^s^Cat-Ver)	156.0 ± 4.2	180	34.1 ± 0.7	0.52 ± 0.03
**8**	Ph_2_Ge(4,6-^s^Cat-Het)	12.0 ± 0.5	57	29.2 ± 0.9	0.58 ± 0.02
**9**	Ph_2_Ge(4,6-Cat-PhOH)	7.5 ± 0.3	50	35.2 ± 0.5	0.42 ± 0.08
**10**	Ph_2_Ge(3,5-Cat)	130.4 ± 2.0	150	45.2 ± 0.9	0.35 ± 0.03
	Trolox	12.0 ± 0.50	10	16.0 ± 1.0	1.00 ± 0.03
	^a^ Ph_3_Sb(4,6-^s^Cat-Bu)	29.5 ± 1.20	170	-	-
	^a^ Ph_3_Sb(3,6-Cat)	17.3 ± 0.50	60	-	-

^a^ Data for triphenylantimony(V) catecholates are from refs. [85,92].

## Data Availability

The original contributions presented in the study are included in the article/Supplementary Materials, further inquiries can be directed to the corresponding author/s.

## Supplementary Materials

The following supporting information can be downloaded at https://www.mdpi.com/article/10.3390/ijms25169011/s1.
