# Supplementary material for: Synthesis, Structure, Electrochemical Properties, and Antioxidant Activity of Organogermanium(IV) Catecholate Complexes
Source: Int J Mol Sci. 2024 Aug 19;25(16):9011. doi: 10.3390/ijms25169011 (PMC11354772; doi:10.3390/ijms25169011)
Supplement: Supplementary file 1 [file ijms-25-09011-s001.zip › ijms-3142158-supplementary.pdf]

Supplementary Information

Synthesis, Structure, Electrochemical Properties, and Antioxidant Activity of Organogermanium(IV) Catecholate Complexes

Daria A. Burmistrova, Nadezhda P. Pomortseva, Yulia K. Voronina, Mikhail A. Kiskin, Fedor M. Dolgushin, Nadezhda T. Berberova, Igor L. Eremenko, Andrey I. Poddel’sky, Ivan V. Smolyaninov

Content

Figure S1. The ^{1}H NMR spectrum of Et_2Ge(3,6-Cat) (**1**) in $CDCl_3$. S2
Figure S2. The $^{13}C\{^1H\}$ NMR spectrum of Et_2Ge(3,6-Cat) (**1**) in $CDCl_3$. S2
Figure S3. The ^{1}H NMR spectrum of Ph_2Ge(3,6-Cat) (**2**) in $CDCl_3$. S3
Figure S4. The $^{13}C\{^1H\}$ NMR spectrum of Ph_2Ge(3,6-Cat) (**2**) in $CDCl_3$. S3
Figure S5. The ^{1}H NMR spectrum of Ph_2Ge(4,6-^{s}Cat-tBu) (**3**) in $CDCl_3$. S4
Figure S6. The $^{13}C\{^1H\}$ NMR spectrum Ph_2Ge(4,6-^{s}Cat-tBu) (**3**) in $CDCl_3$. S4
Figure S7. The ^{1}H NMR spectrum of Ph_2Ge(4,6-^{s}Cat-Ad) (**4**) in $CDCl_3$. S5
Figure S8. The $^{13}C\{^1H\}$ NMR spectrum of Ph_2Ge(4,6-^{s}Cat-Ad) (**4**) in $CDCl_3$. S5
Figure S9. The ^{1}H NMR spectrum of Ph_2Ge(4,6-^{s}Cat-BuOH) (**5**) in $CDCl_3$. S6
Figure S10. The $^{13}C\{^1H\}$ NMR spectrum of Ph_2Ge(4,6-^{s}Cat-BuOH) (**5**) in $CDCl_3$. S6
Figure S11. The ^{1}H NMR spectrum of Ph_2Ge(4,6-^{s}Cat-Fur) (**6**) in $CDCl_3$. S7
Figure S12. The $^{13}C\{^1H\}$ NMR spectrum of Ph_2Ge(4,6-^{s}Cat-Fur) (**6**) in $CDCl_3$. S7
Figure S13. The ^{1}H NMR spectrum of Ph_2Ge(4,6-^{s}Cat-Ver) (**7**) in $CDCl_3$. S8
Figure S14. The $^{13}C\{^1H\}$ NMR spectrum of Ph_2Ge(4,6-^{s}Cat-Ver) (**7**) in $CDCl_3$. S8
Figure S15. The ^{1}H NMR spectrum of Ph_2Ge(4,6-^{s}Cat-Het) (**8**) in $CDCl_3$. S9
Figure S16. The $^{13}C\{^1H\}$ NMR spectrum of Ph_2Ge(4,6-^{s}Cat-Het) (**8**) in $CDCl_3$. S9
Figure S17. The ^{1}H NMR spectrum of Ph_2Ge(4,6-Cat-PhOH) (**9**) in $CDCl_3$. S10
Figure S18. The $^{13}C\{^1H\}$ NMR spectrum of Ph_2Ge(4,6-Cat-PhOH) (**9**) in $CDCl_3$. S10
Figure S19. The CV curve of the oxidation of complex Ph_2Ge(3,5-Cat) (**10**). S11
Figure S20. The CV curve of the reduction of complex Et_2Ge(3,6-Cat)(**1**) after electrolysis. S11
Figure S21. The CV curves of oxidation of complex Et_2Ge(3,6-Cat) (**1**) in MeCN. S12
Figure S22. The CV curve of oxidation of complex Ph_2Ge(3,6-Cat) (**2**) in MeCN. S12
Figure S23. The CV curves of the oxidation of complex Ph_2Ge(4,6-^{s}Cat-tBu) (**3**). S13
Figure S24. The CV curve of oxidation of complex Ph_2Ge(4,6-^{s}Cat-Ad) (**4**). S13
Figure S25. The CV curve of oxidation of complex Ph_2Ge(4,6-^{s}Cat-Ver) (**7**). S14
Figure S26. The CV curves of oxidation of complex Ph_2Ge(4,6-^{s}Cat-Fur) (**6**). S14
Figure S27. The CV curves of oxidation of complex Ph_2Ge(4,6-Cat-PhOH) (**9**). S15
Table S1. Crystal data and structure refinement for **1**, **2**, **3**, **6**, and **8**. S16

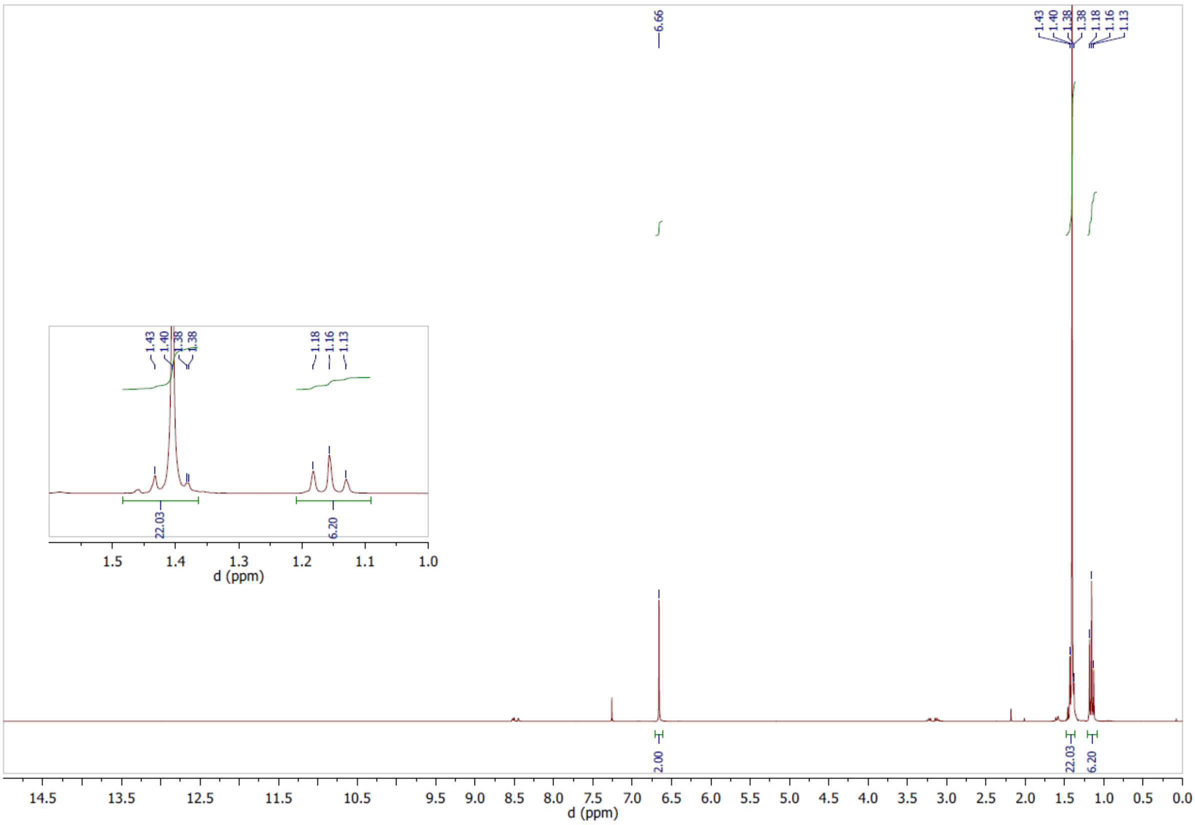

Figure S1. The ^{1}H NMR spectrum of complex Et_2Ge(3,6-Cat) (**1**) in $CDCl_3$.

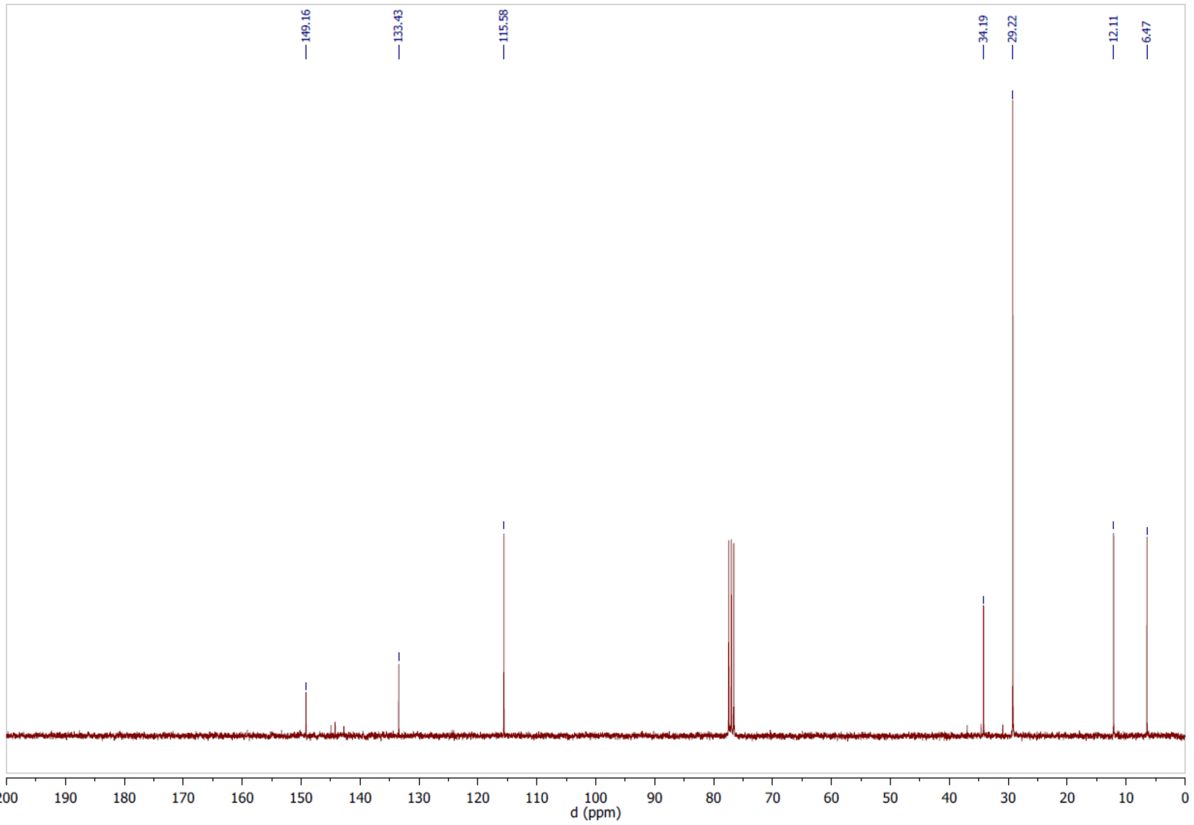

Figure S2. The $^{13}C\{^{1}H\}$ NMR spectrum of complex Et_2Ge(3,6-Cat) (**1**) in $CDCl_3$.

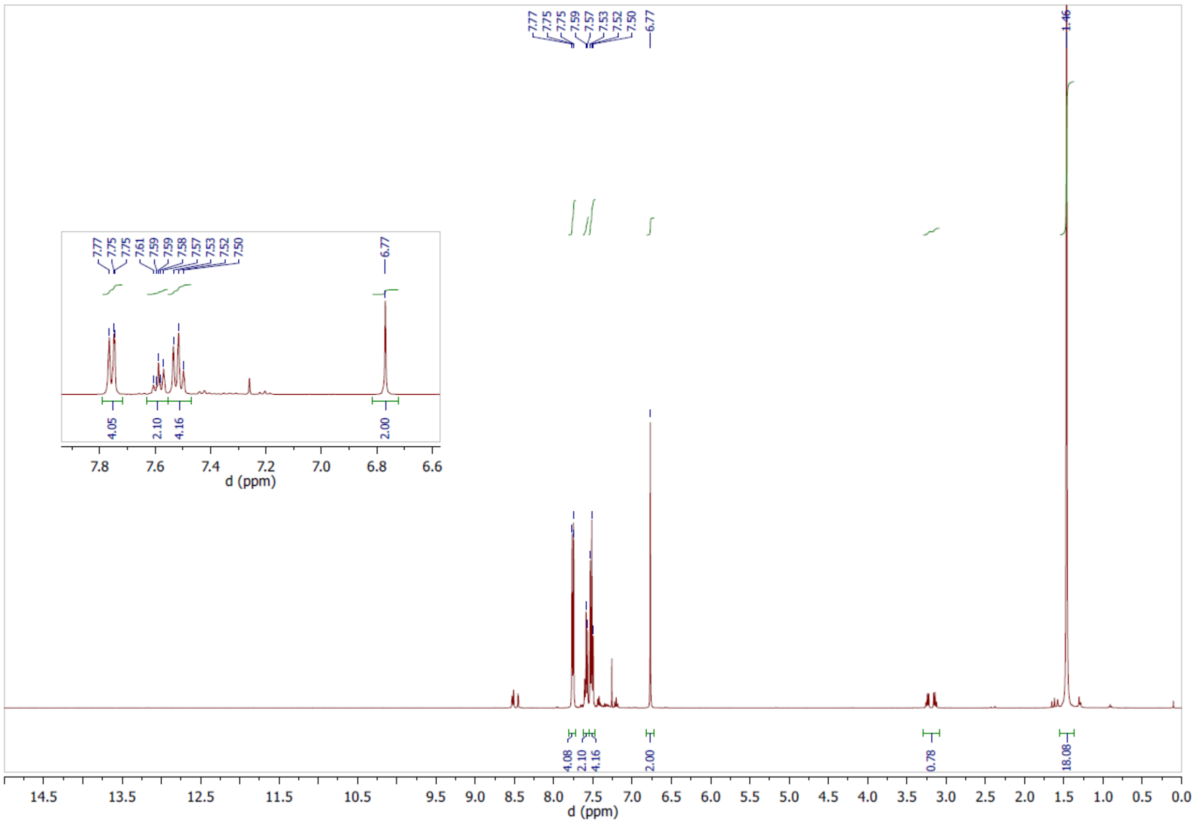

Figure S3. The ^{1}H NMR spectrum of complex Ph_2Ge(3,6-Cat) (**2**) in $CDCl_3$.

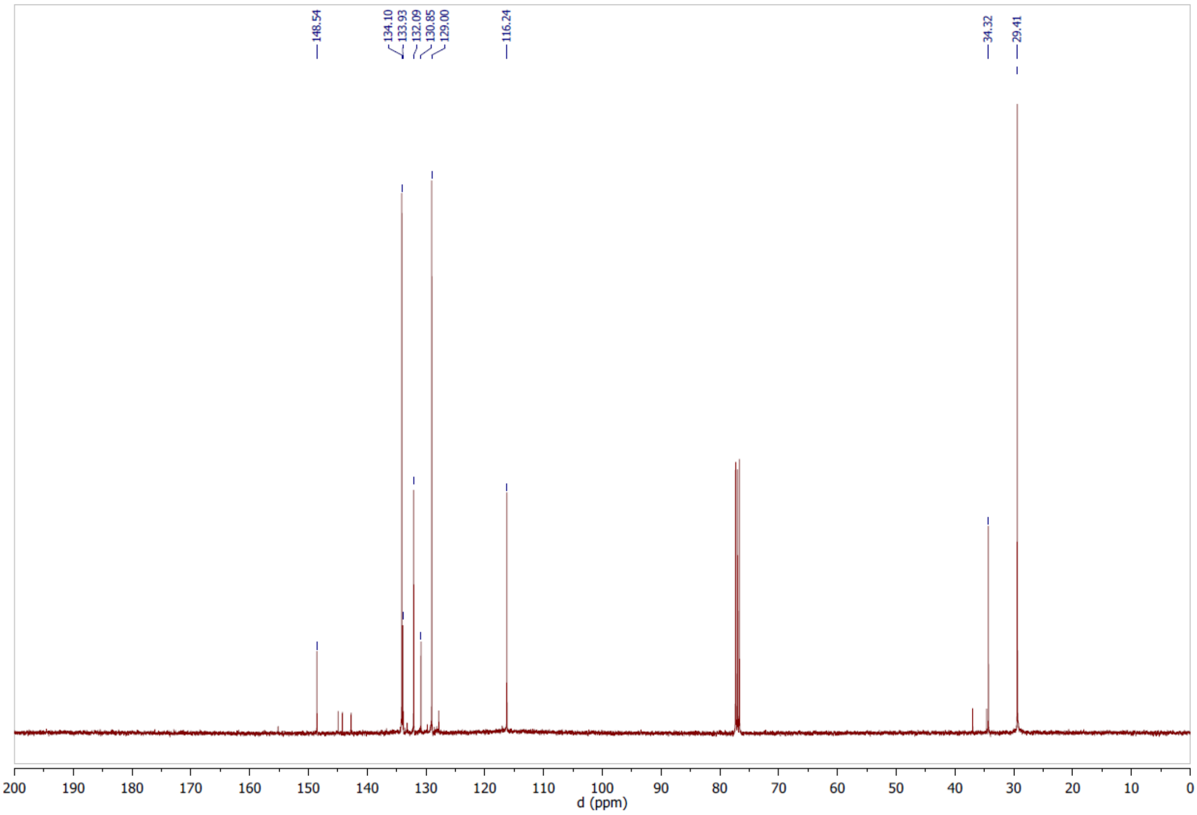

Figure S4. The $^{13}C\{^{1}H\}$ NMR spectrum of complex Ph_2Ge(3,6-Cat) (**2**) in $CDCl_3$.

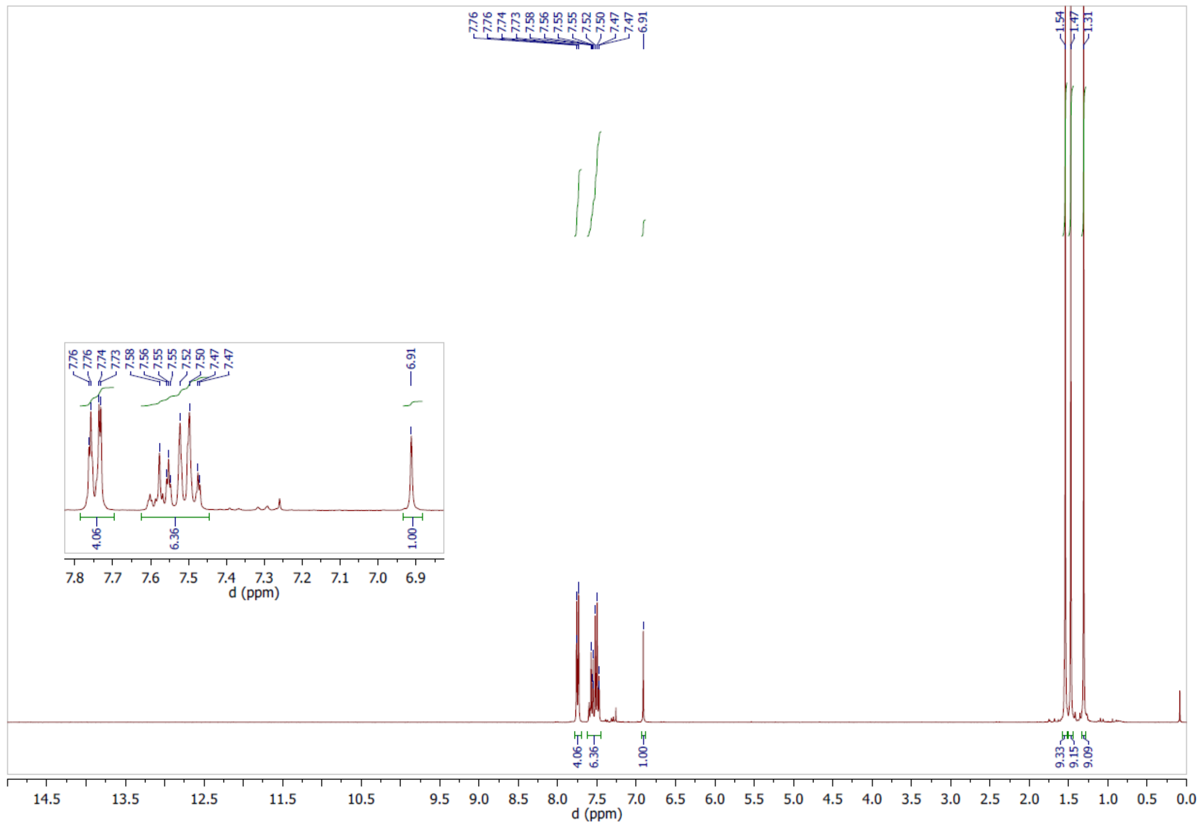

Figure S5. The ^{1}H NMR spectrum of complex Ph_2Ge(4,6-[S]Cat-tBu) (**3**) in $CDCl_3$.

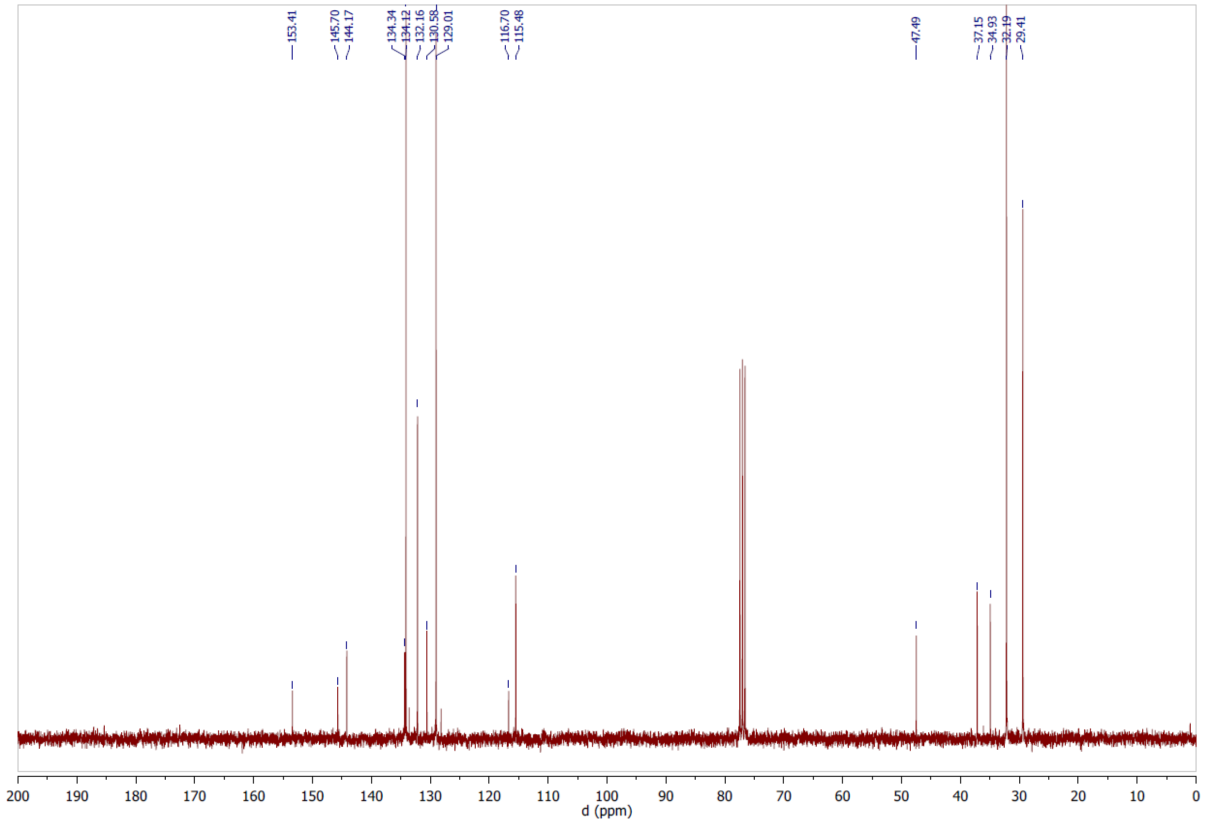

Figure S6. The ^{13}C{^{1}H} NMR spectrum of complex Ph_2Ge(4,6-[S]Cat-tBu) (**3**) in $CDCl_3$.

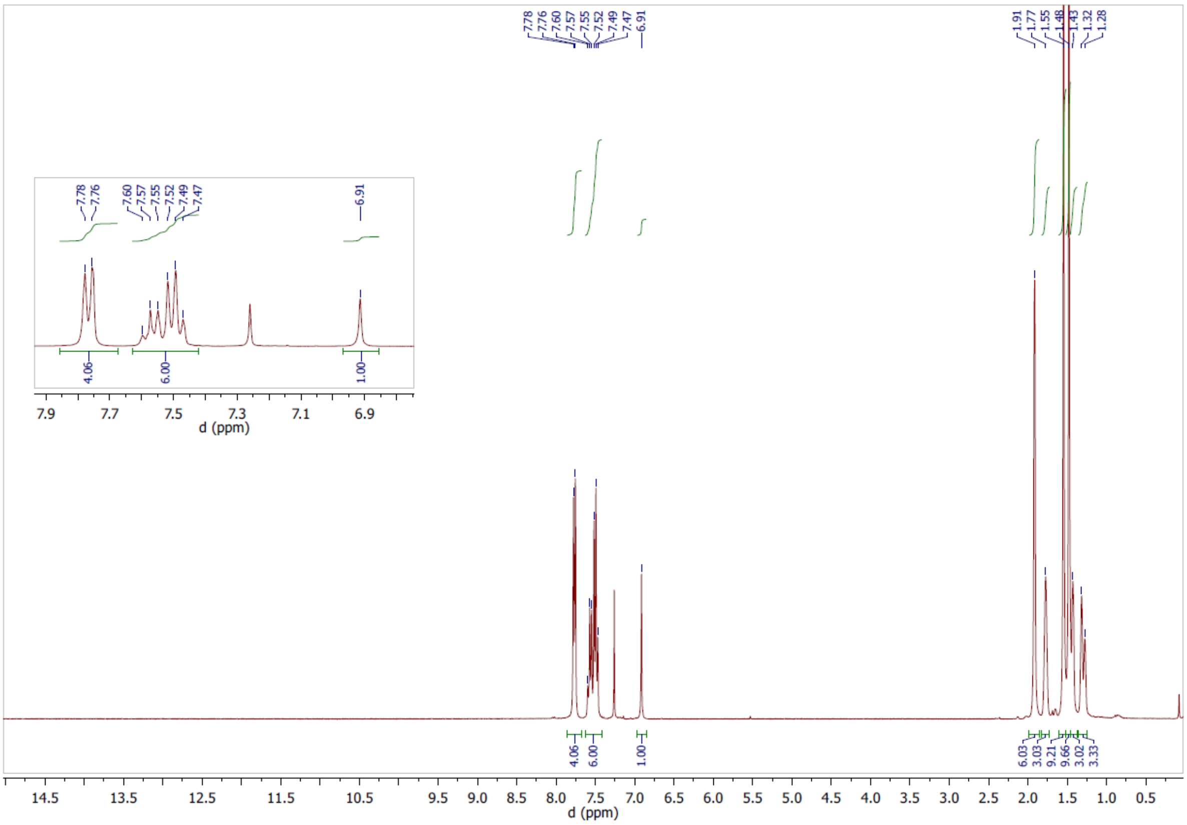

Figure S7. The ^{1}H NMR spectrum of complex Ph_2Ge(4,6-^{s}Cat-Ad) (**4**) in $CDCl_3$.

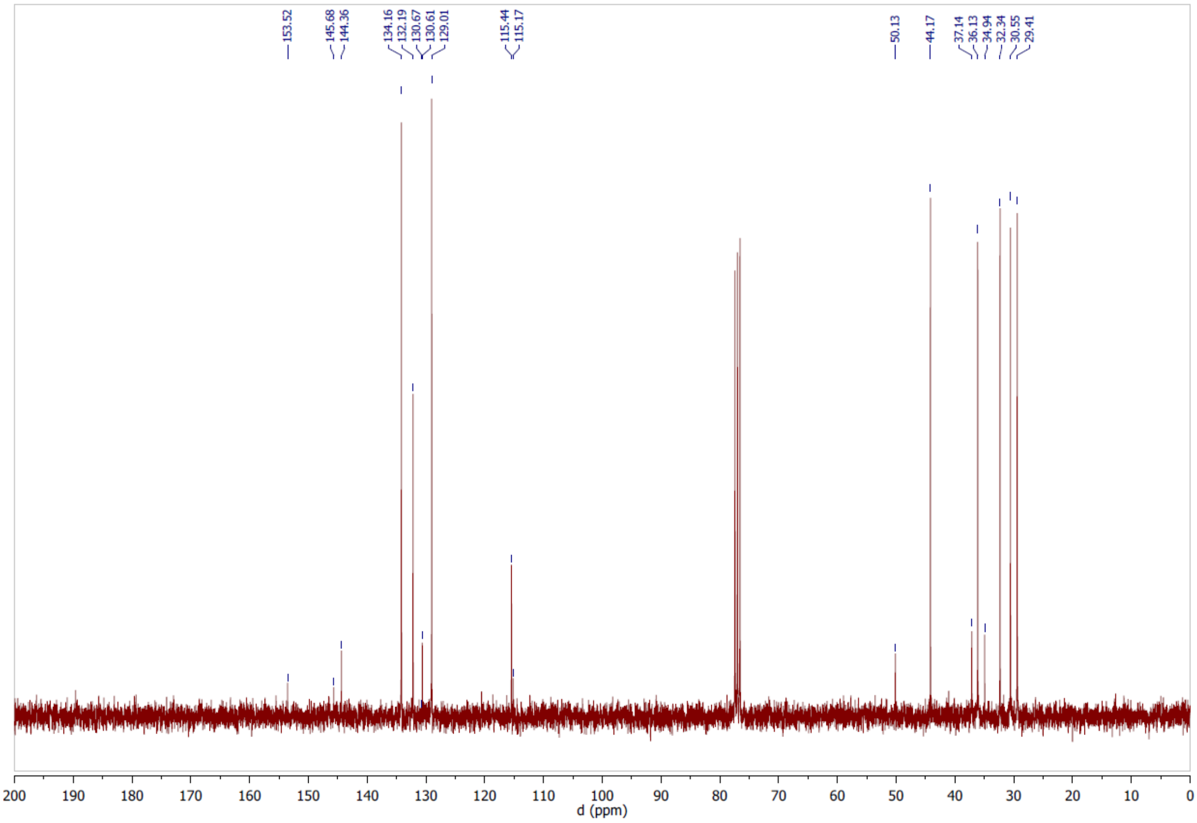

Figure S8. The $^{13}C\{^{1}H\}$ NMR spectrum of complex Ph_2Ge(4,6-^{s}Cat-Ad) (**4**) in $CDCl_3$.

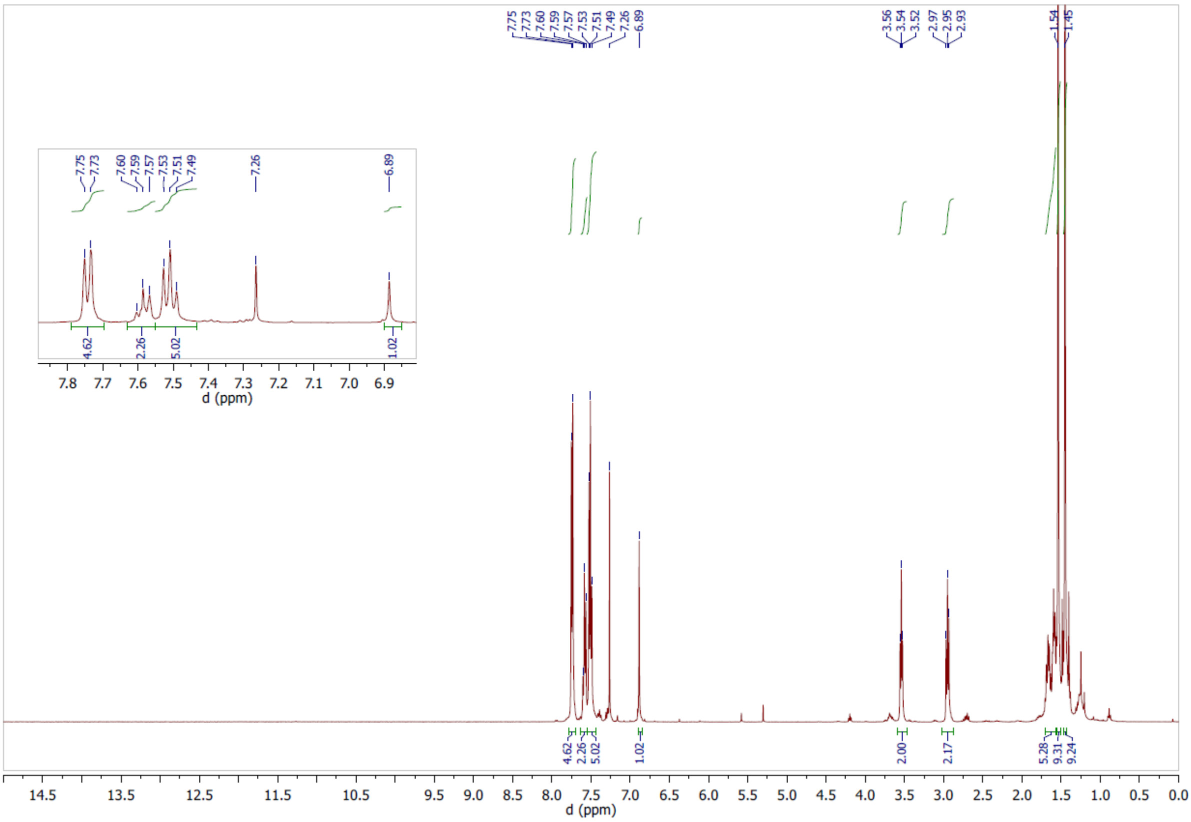

Figure S9. The ^{1}H NMR spectrum of complex Ph_2Ge(4,6-[S]Cat-BuOH) (**5**) in $CDCl_3$.

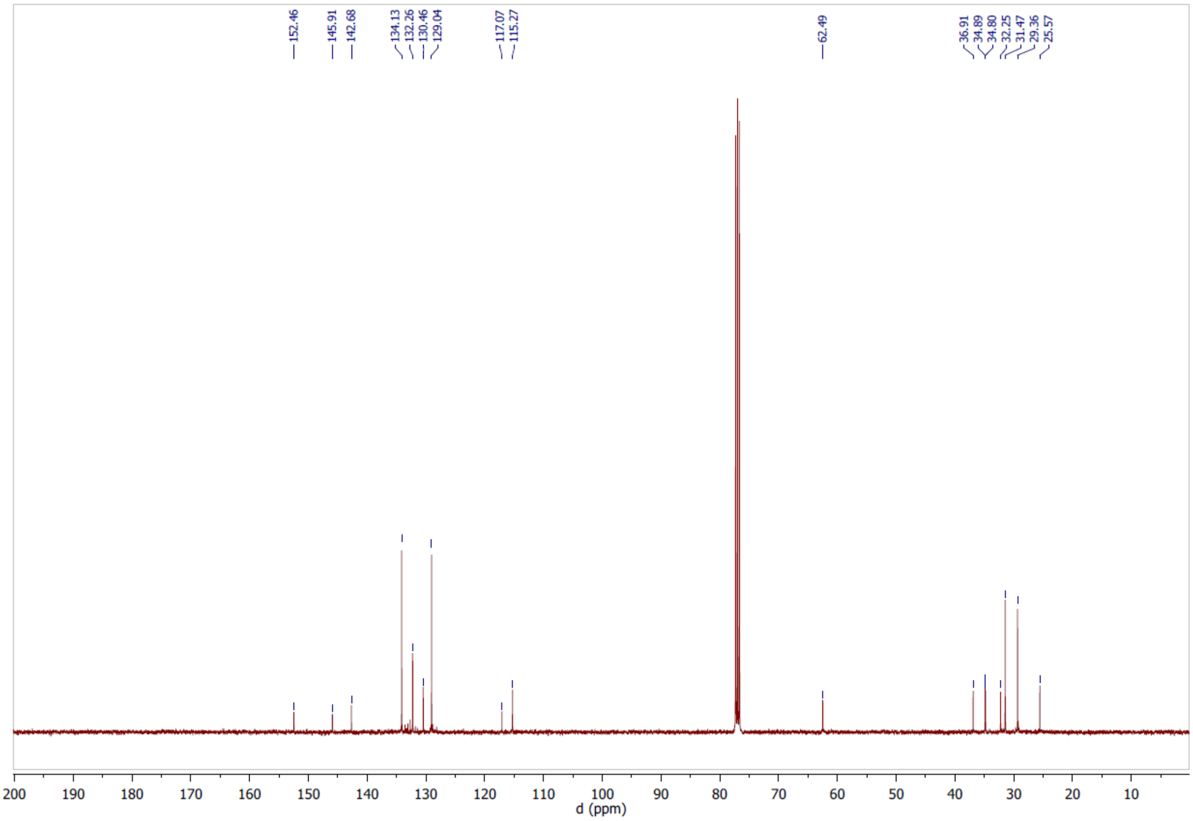

Figure S10. The ^{13}C{^{1}H} NMR spectrum of complex Ph_2Ge(4,6-[S]Cat-BuOH) (**5**) in $CDCl_3$.

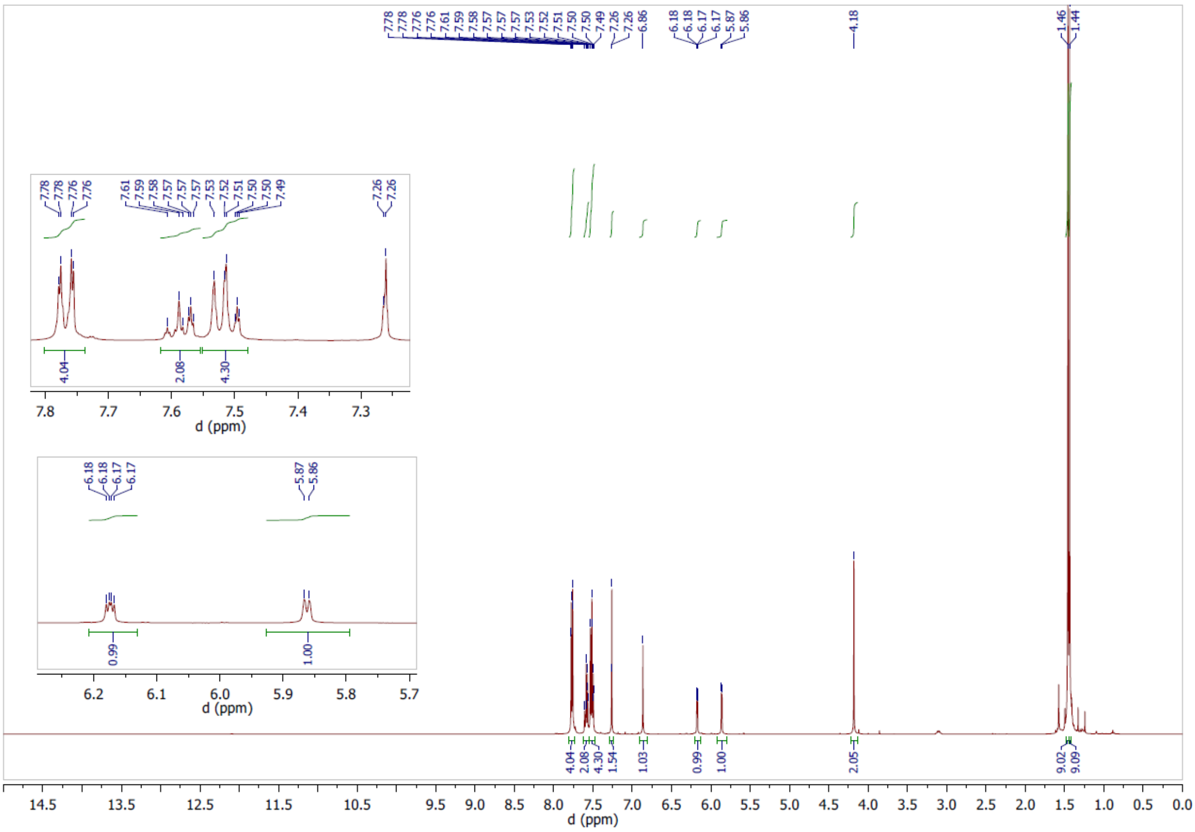

Figure S11. The ^{1}H NMR spectrum of complex Ph_2Ge(4,6-[S]Cat-Fur) (**6**) in $CDCl_3$.

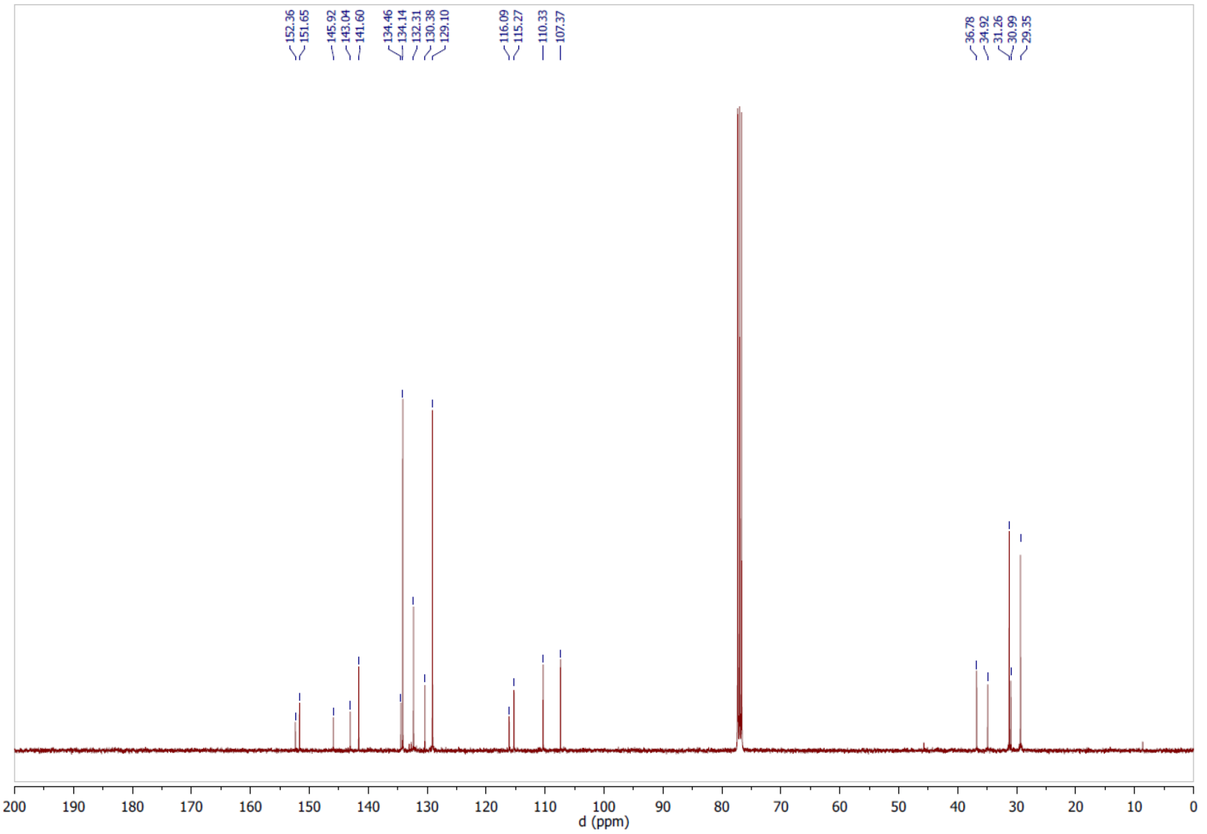

Figure S12. The ^{13}C{^{1}H} NMR spectrum of complex Ph_2Ge(4,6-[S]Cat-Fur) (**6**) in $CDCl_3$.

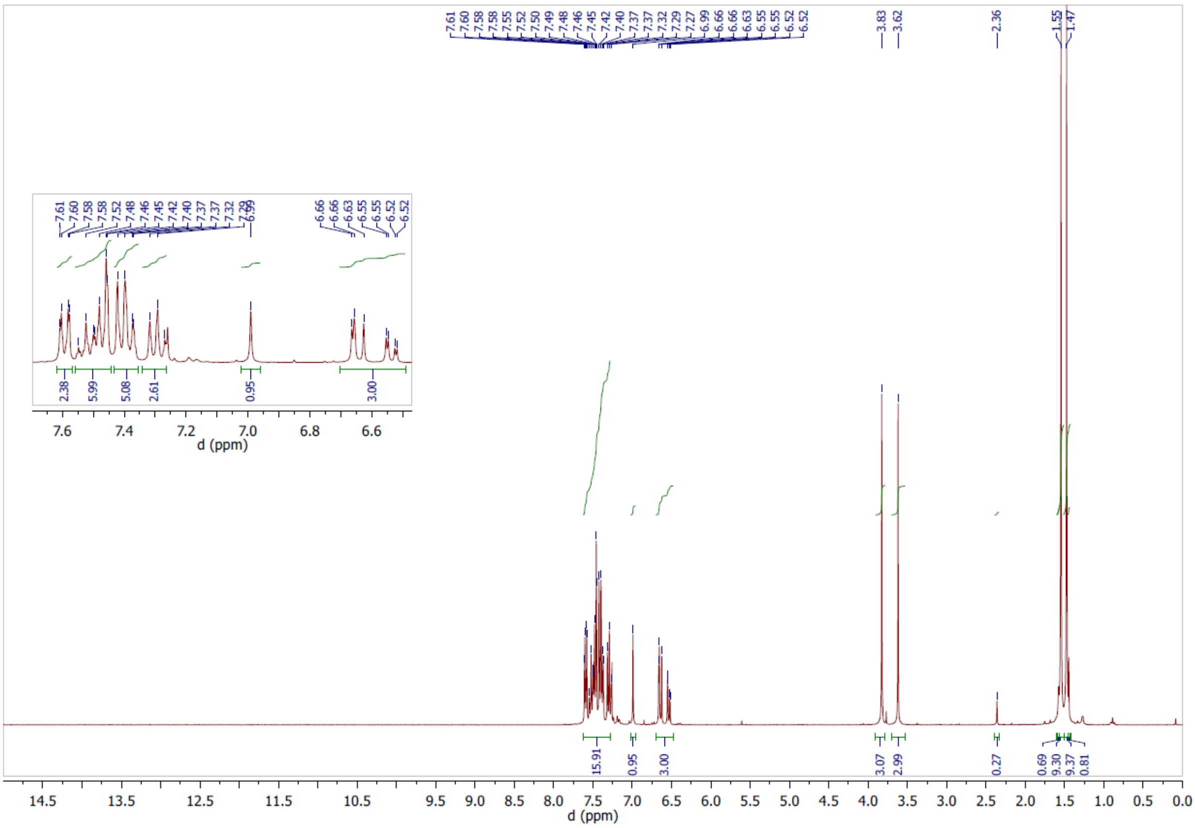

Figure S13. The ^{1}H NMR spectrum of $Ph_2Ge(4,6\text{-}^{s}Cat\text{-}Ver)$ (**7**) in $CDCl_3$.

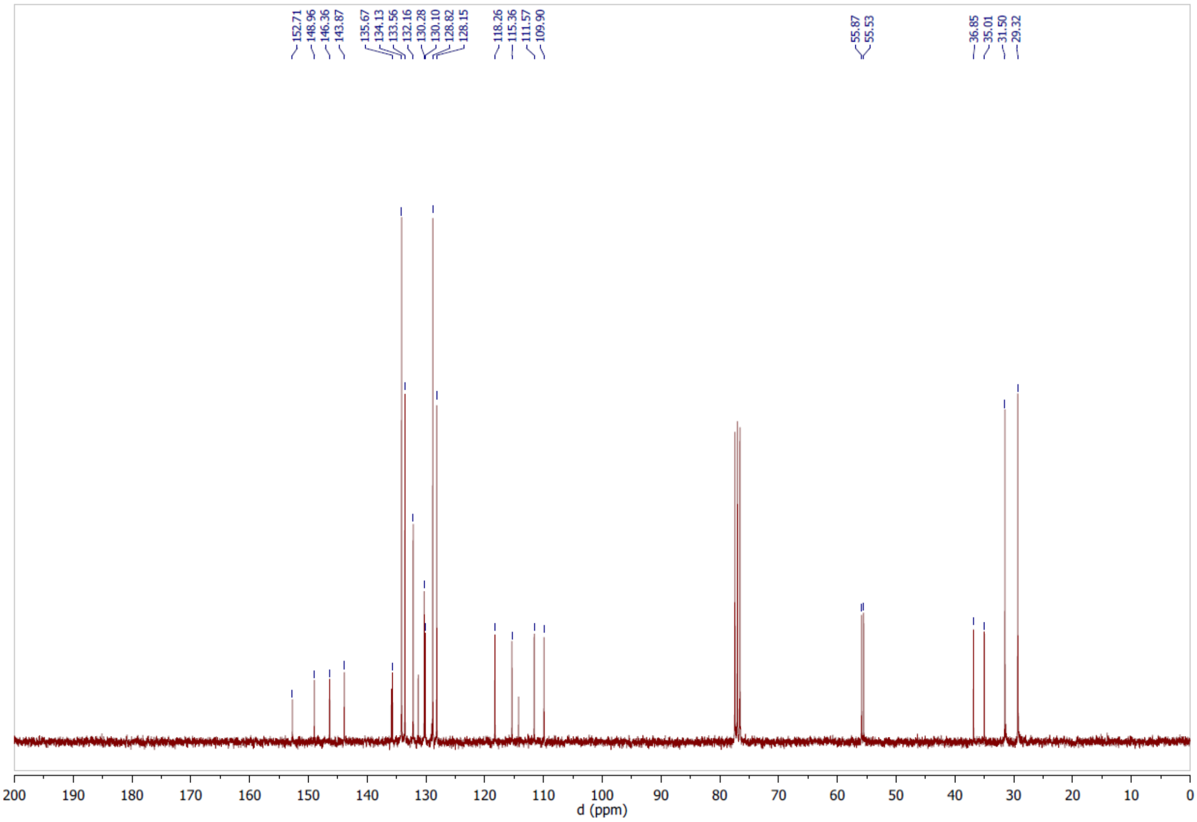

Figure S14. The $^{13}C\{^{1}H\}$ NMR spectrum of $Ph_2Ge(4,6\text{-}^{s}Cat\text{-}Ver)$ (**7**) in $CDCl_3$.

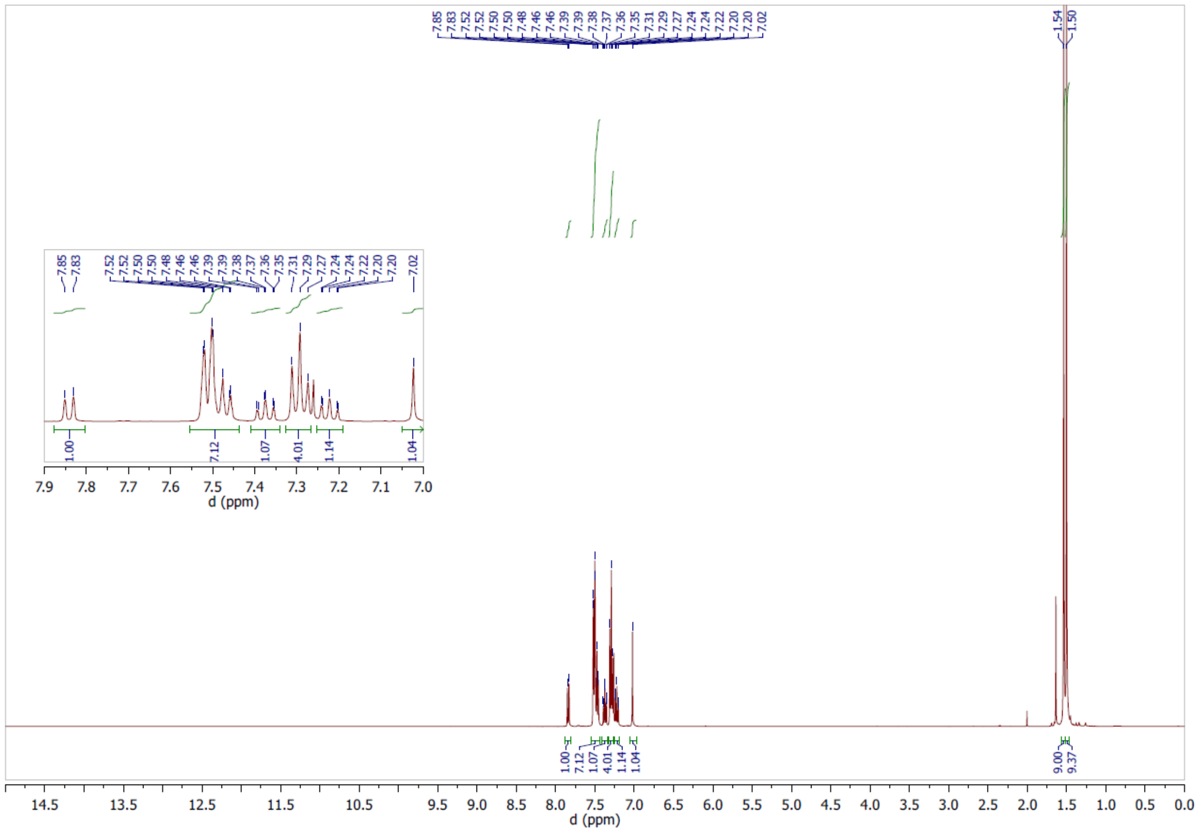

Figure S15. The ^{1}H NMR spectrum of $Ph_2Ge(4,6\text{-}^{S}Cat\text{-}Het)$ (**8**) in $CDCl_3$.

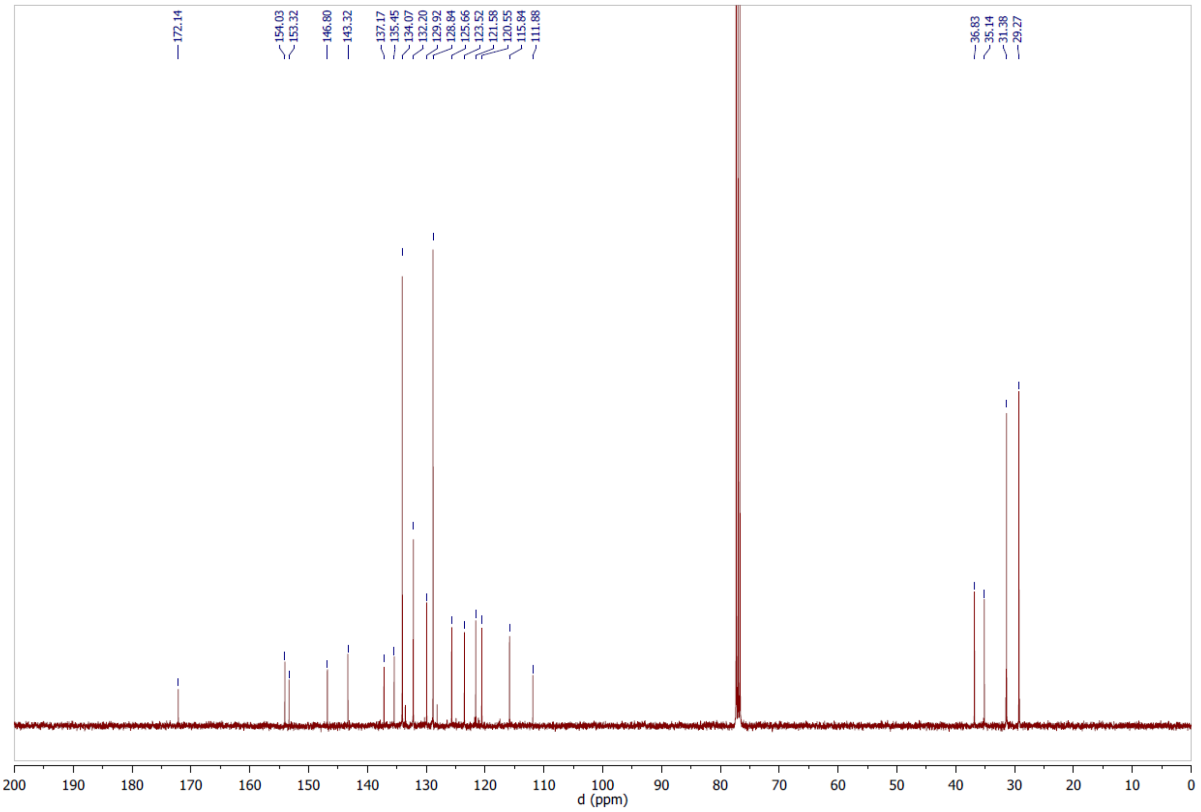

Figure S16. The $^{13}C\{^{1}H\}$ NMR spectrum of $Ph_2Ge(4,6\text{-}^{S}Cat\text{-}Het)$ (**8**) in $CDCl_3$.

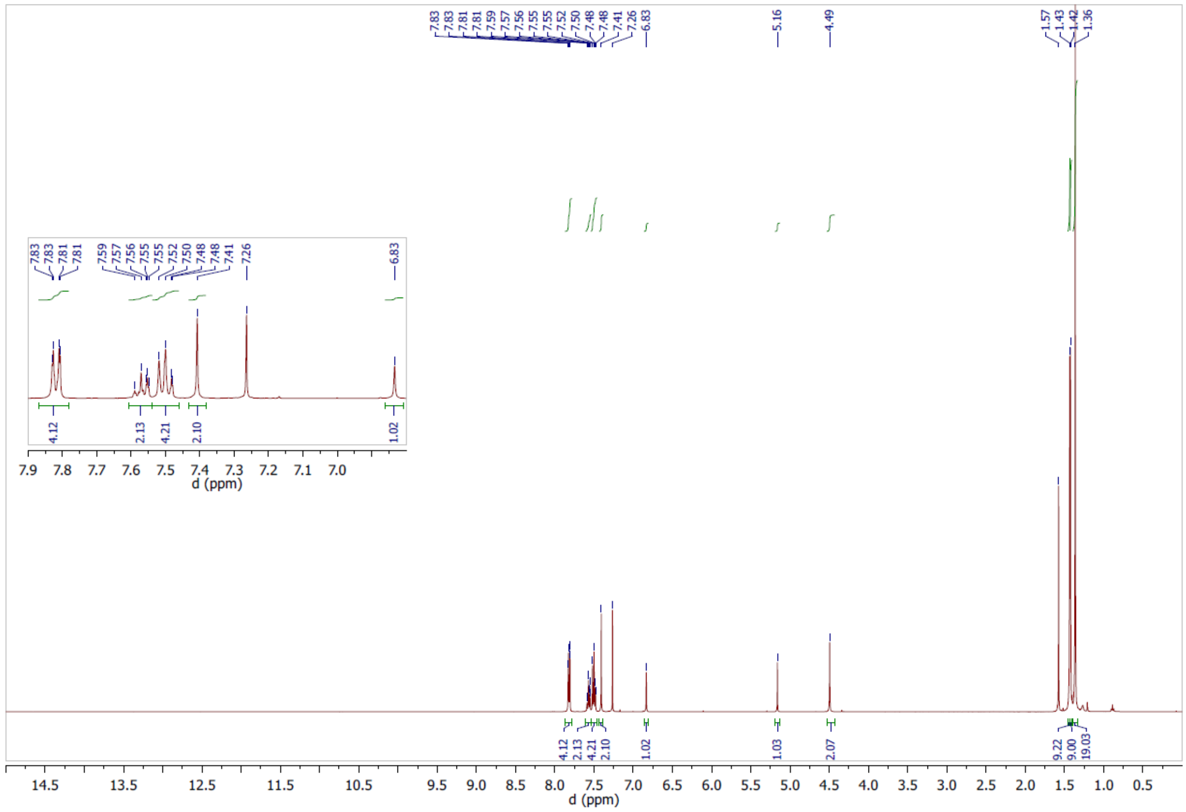

Figure S17. The ^{1}H NMR spectrum of Ph_2Ge(4,6-Cat-PhOH) (**9**) in $CDCl_3$.

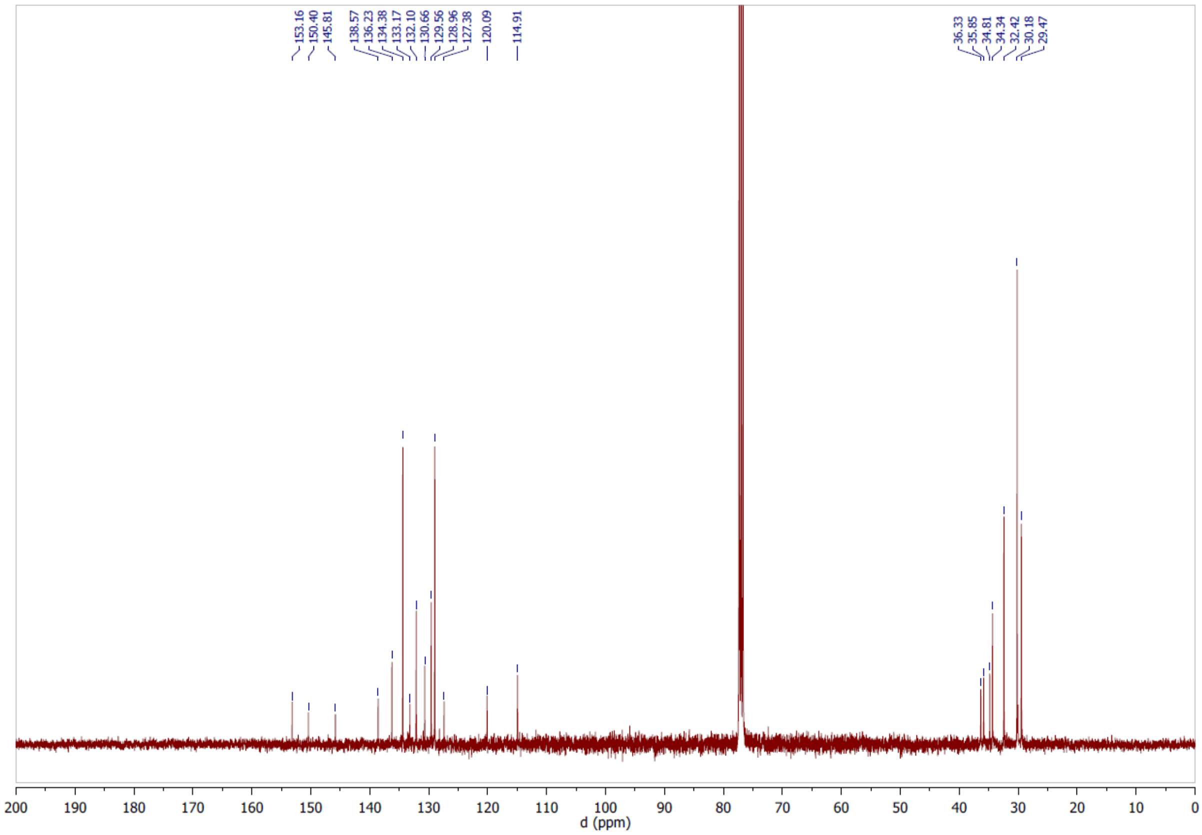

Figure S18. The $^{13}C\{^{1}H\}$ NMR spectrum of Ph_2Ge(4,6-Cat-PhOH) (**9**) in $CDCl_3$.

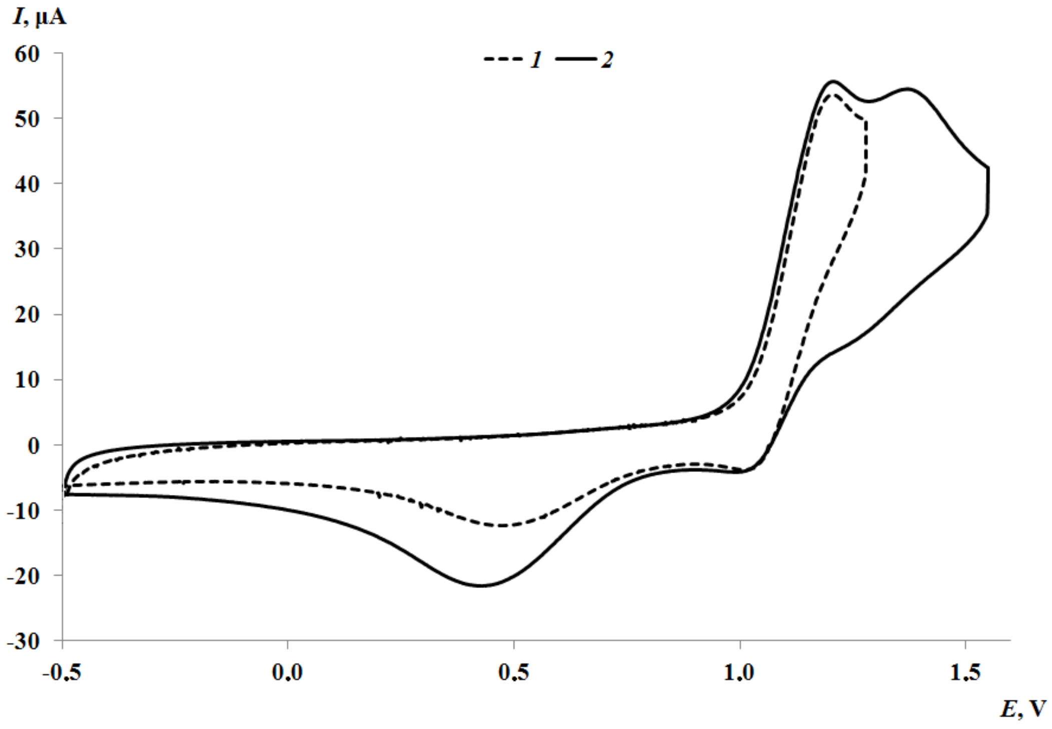

Figure S19. The CV curves of the oxidation of complex Ph_2Ge(3,5-Cat) (**10**) in potential ranges from -0.50 to 1.28 V (curve 1); from -0.50 to 1.55 V (curve 2) (CH_2Cl_2, GC anode, Ag/AgCl/KCl(sat.), 0.15M nBu_4NClO_4, C = $3{\cdot}10^{-3}$ M, argon).

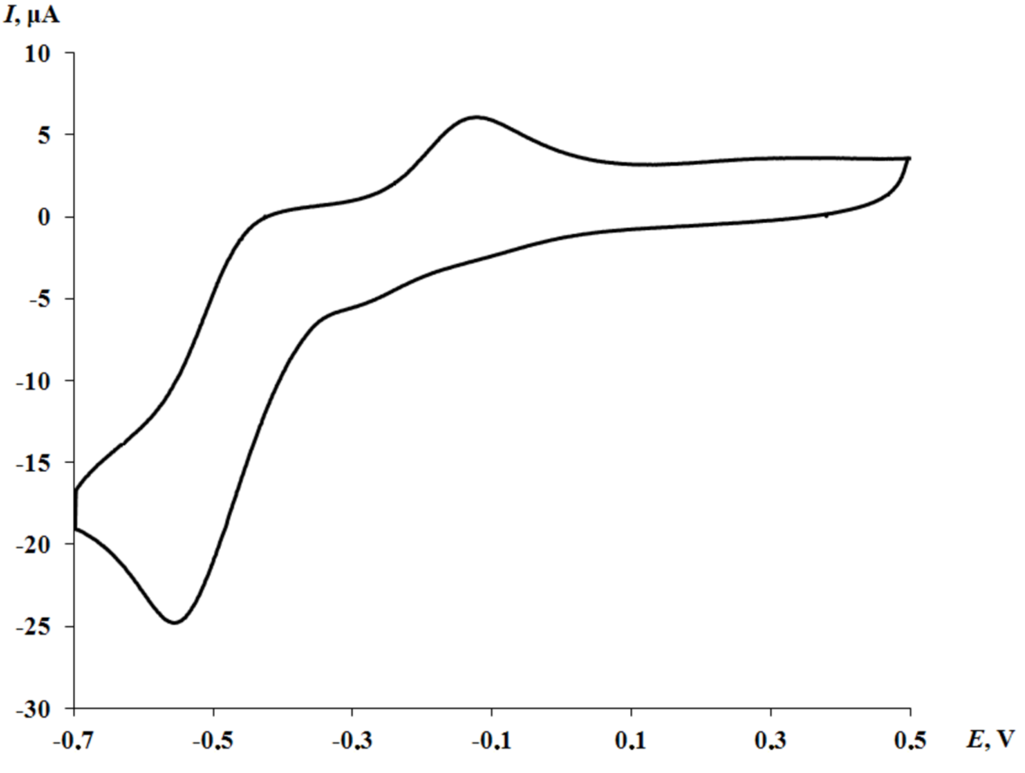

Figure S20. The CV curve of the reduction of complex Et_2Ge(3,6-Cat) (**1**) after electrolysis under 1.25 V (1.5 h) in potential range from 0.50 to -0.70 V (CH_2Cl_2, GC anode, Ag/AgCl/KCl(sat.), 0.15M nBu_4NClO_4, C = $2{\cdot}10^{-3}$ M, argon).

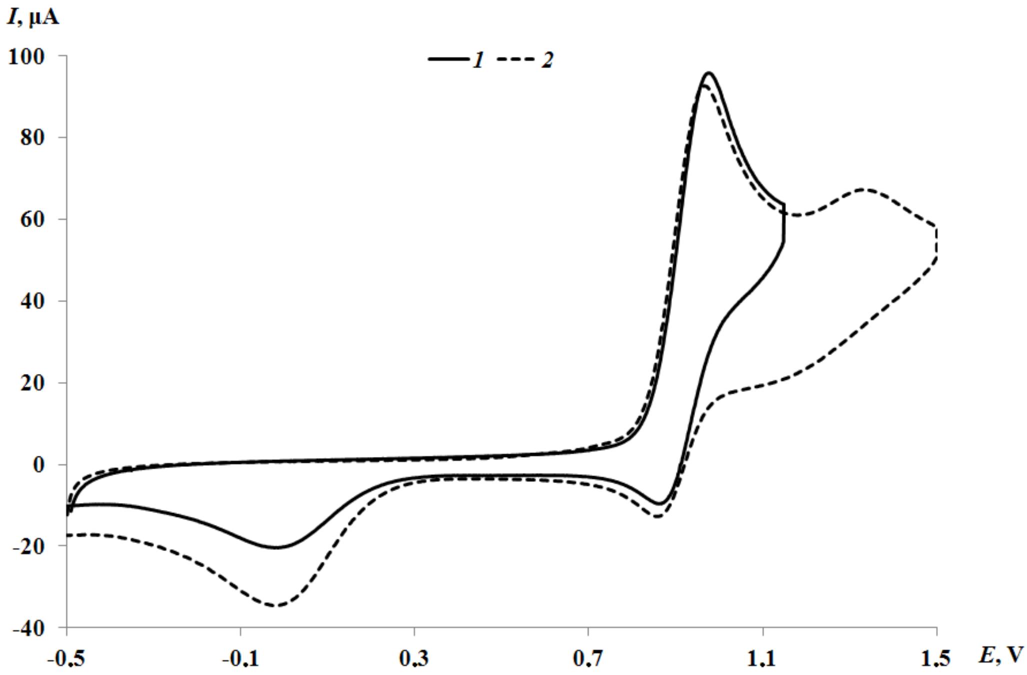

Figure S21. The CV curves of oxidation of complex Et_2Ge(3,6-Cat) (**1**) in potential in potential ranges from -0.50 to 1.15 V (curve 1); from -0.50 to 1.50 V (curve 2) (CH_3CN, GC anode, Ag/AgCl/KCl(sat.), 0.15M nBu_4NClO_4, C = $3\cdot10^{-3}$ M, argon).

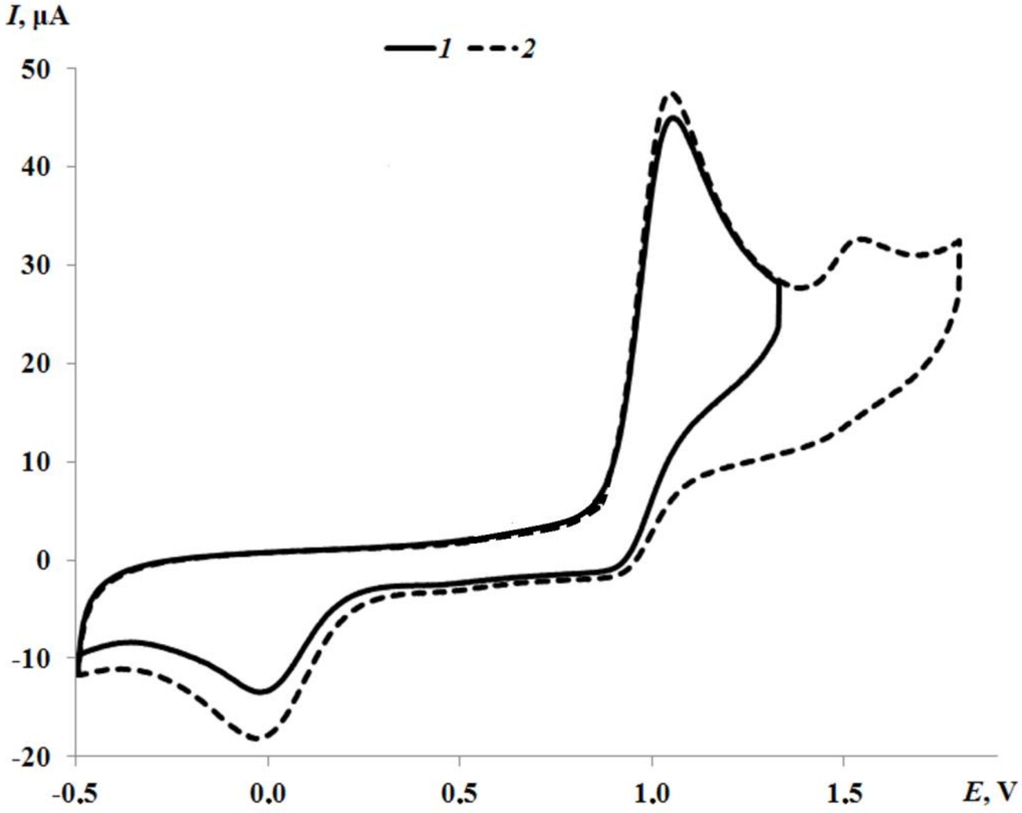

Figure S22. The CV curves of oxidation of Ph_2Ge(3,6-Cat) (**2**) in potential ranges from -0.50 to 1.32 V (curve 1); from -0.50 to 1.80 V (curve 2) (CH_3CN, GC anode, Ag/AgCl/KCl(sat.), 0.15M nBu_4NClO_4, C = $1.5\cdot10^{-3}$ M, argon).

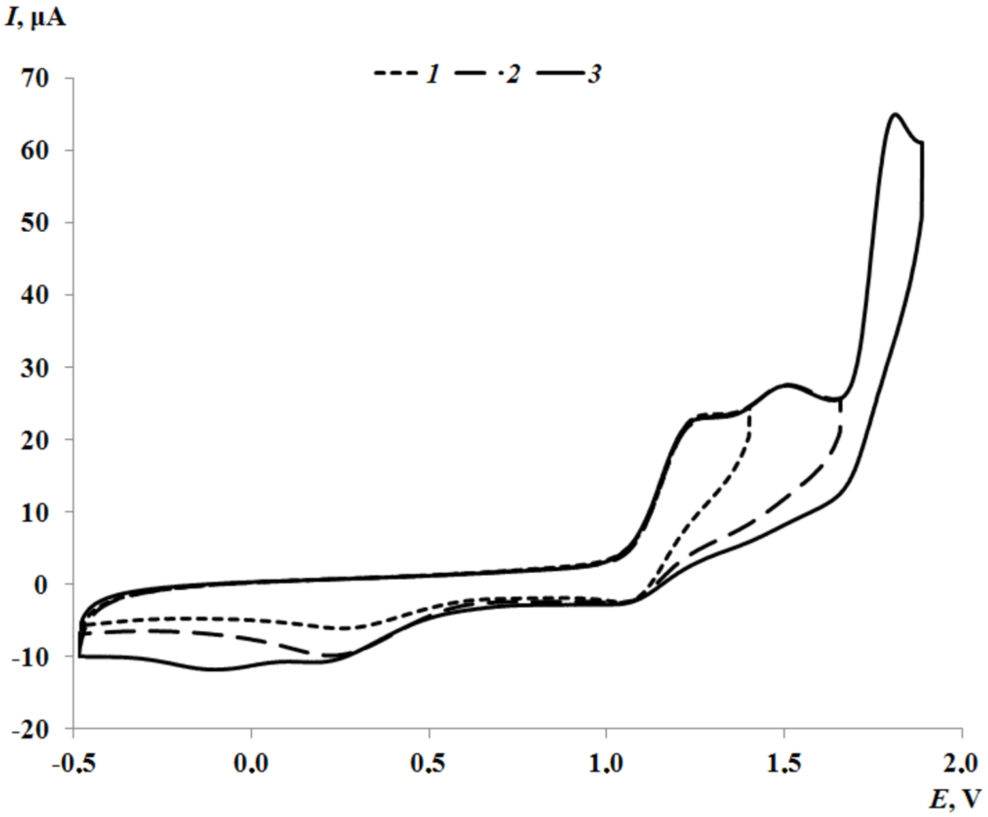

Figure S23. The CV curves of oxidation of complex Ph_2Ge(4,6-^{S}Cat-tBu) (**3**) in potential ranges from -0.50 to 1.40 V (curve 1); from -0.50 to 1.65 V (curve 2); from -0.50 to 1.89 V (curve 3) (CH_2Cl_2, GC anode, Ag/AgCl/KCl(sat.), 0.15M nBu_4NClO_4, C = $1.0 \cdot 10^{-3}$ M, argon).

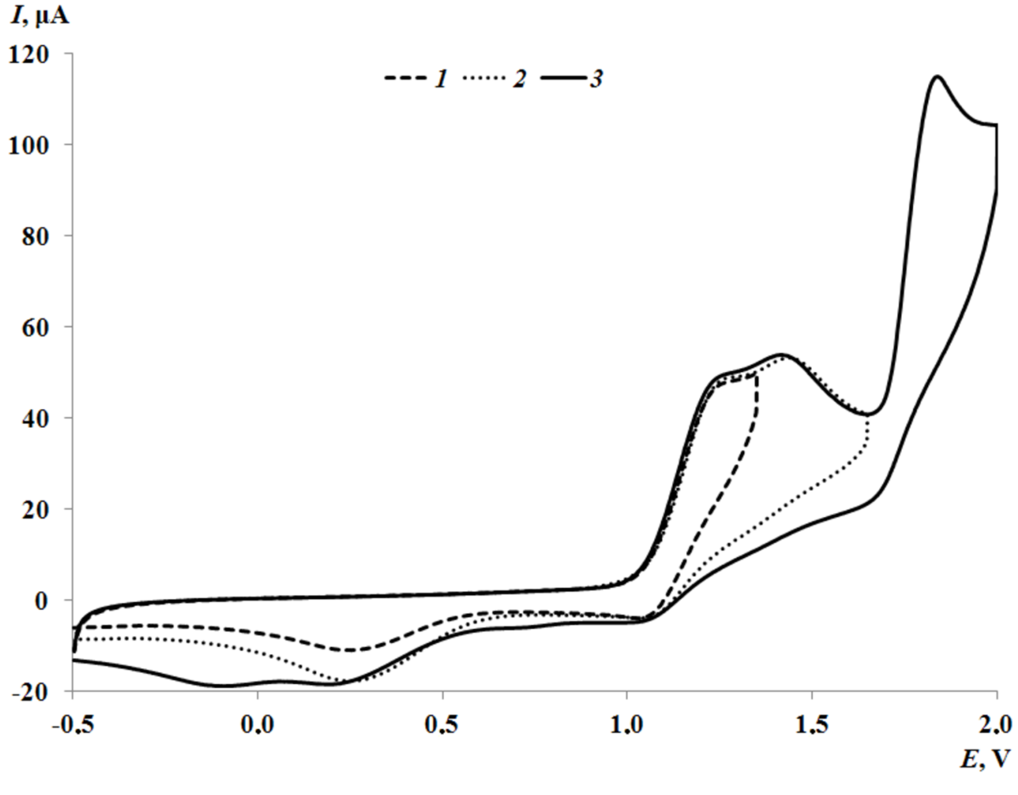

Figure S24. The CV curves of oxidation of complex Ph_2Ge(4,6-^{S}Cat-Ad) (**4**) in potential ranges from -0.50 to 1.35 V (curve 1); from -0.50 to 1.65 V (curve 2); from -0.50 to 2.00 V (curve 3) (CH_2Cl_2, GC anode, Ag/AgCl/KCl(sat.), 0.15M nBu_4NClO_4, C = $1.0 \cdot 10^{-3}$ M, argon).

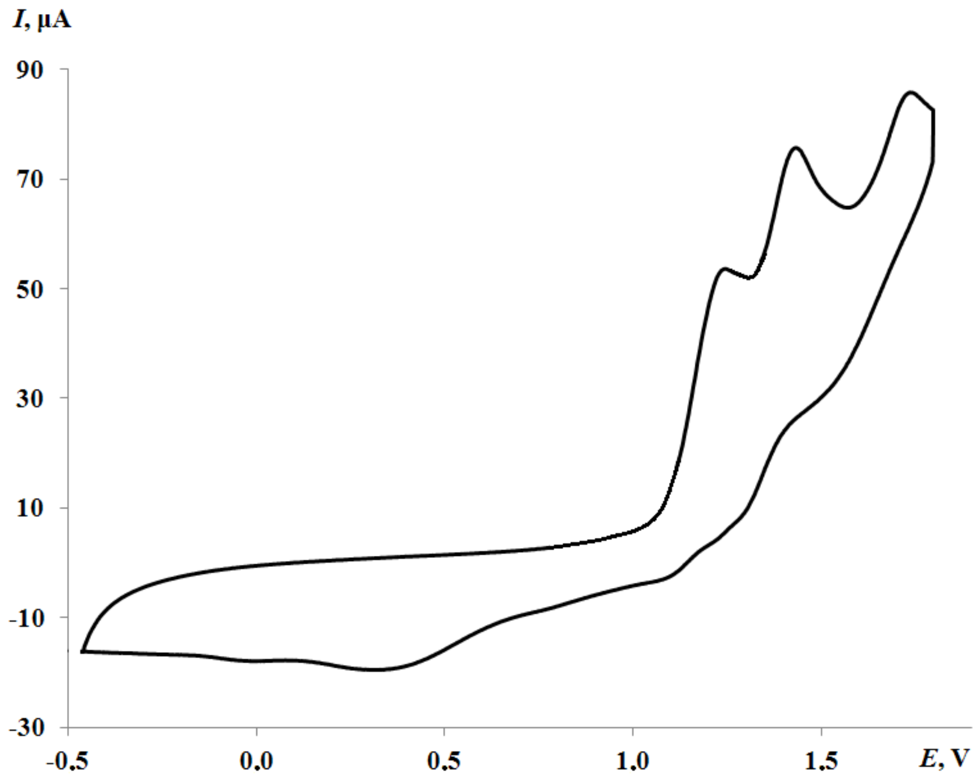

Figure S25. The CV curve of the oxidation of complex Ph_2Ge(4,6-^{S}Cat-Ver) (**7**) in potential range from -0.45 to 1.80 V (CH_2Cl_2, GC anode, Ag/AgCl/KCl(sat.), 0.15M nBu_4NClO_4, C = $3 \cdot 10^{-3}$ M, argon).

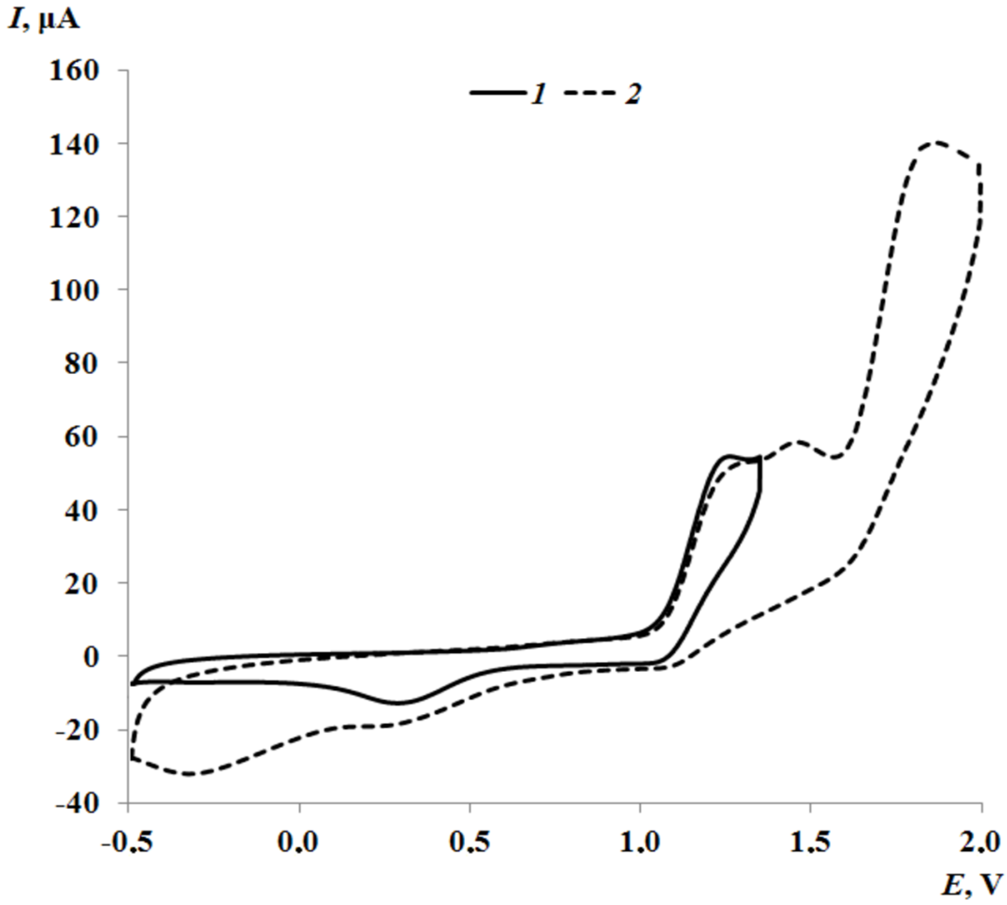

Figure S26. The CV curves of oxidation of complex Ph_2Ge(4,6-^{S}Cat-Fur) (**6**) in potential ranges from -0.50 to 1.35 V (curve 1); from -0.50 to 2.00 V (curve 2) (CH_2Cl_2, GC anode, Ag/AgCl/KCl(sat.), 0.15M nBu_4NClO_4, C = $3.0 \cdot 10^{-3}$ M, argon).

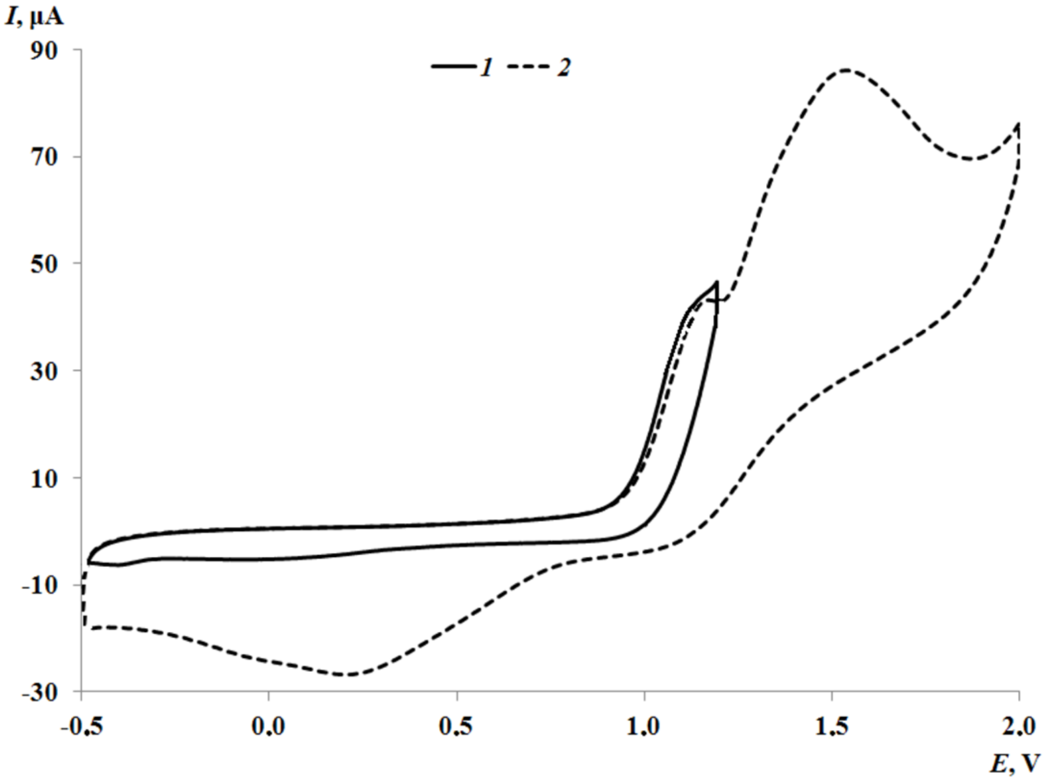

Figure S27. The CV curves of oxidation of complex Ph_2Ge(4,6-Cat-PhOH) (**9**) in potential ranges from -0.50 to 1.20 V (curve 1); from -0.50 to 2.00 V (curve 2) (CH_2Cl_2, GC anode, Ag/AgCl/KCl(sat.), 0.15M nBu_4NClO_4, C = $2.0 \cdot 10^{-3}$ M, argon).

Table S1. Crystal data and structure refinement for **1**, **2**, **3**, **6**, and **8**.

Compound	**1**	**2**	**3**	**6**	**8**
Empirical formula	$C_{18}H_{30}GeO_2$	$C_{26}H_{30}GeO_2$	$C_{30}H_{38}GeO_2S$	$C_{31}H_{34}GeO_3S$	$C_{33}H_{33}GeNO_2S_2$
Formula weight	351.01	447.09	535.25	559.23	612.31
Temperature, K	100(2)	100(2)	100(2)	100(2)	100(2)
Wavelength, Å	0.71073	0.71073	0.71073	0.71073	0.71073
Crystal system	Orthorhombic	Triclinic	Triclinic	Triclinic	Triclinic
space group	Pccn	P-1	P-1	P-1	P-1
Unit cell dimensions					
a, Å	18.0866(5)	9.7762(6)	9.755(3)	9.3204(15)	13.3831(8)
b, Å	19.8786(6)	15.7150(12)	10.179(7)	10.3677(14)	15.1695(8)
c, Å	10.0195(3)	16.5134(11)	16.568(7)	14.934(3)	15.8678(9)
alpha , deg.	90	114.378(2)	94.28(3)	87.256(4)	103.0514(18)
beta, deg.	90	92.784(4)	104.164(15)	82.800(4)	104.9466(19)
gamma, deg.	90	95.748(4)	115.70(3)	77.655(6)	99.4587(19)
Volume, $Å^3$	3602.37(18)	2287.8(3)	1406.3(12)	1398.3(4)	2946.0(3)
Z	8	4	2	2	4
Calculated density, Mg/m^3	1.294	1.298	1.264	1.328	1.381
Absorpt. coeff., mm^{-1}	1.703	1.357	1.187	1.200	1.212
F(000)	1488	936	564	584	1272
Crystal size, mm	0.30 x 0.25 x 0.18	0.24 x 0.158 x 0.12	0.22 x 0.16 x 0.10	0.18 x 0.15 x 0.12	0.48 x 0.36 x 0.22
2Θ range for data collection, deg.	3.045 – 33.169	4.208 – 61.092	2.369 – 33.168	4.022 – 57.996	3.77 – 60.00
Limiting indices	$-17 \leq h \leq 27$<br>$-30 \leq k \leq 30$<br>$-15 \leq l \leq 15$	$-13 \leq h \leq 13$<br>$-22 \leq k \leq 22$<br>$-22 \leq l \leq 23$	$-13 \leq h \leq 14$<br>$-15 \leq k \leq 15$<br>$-25 \leq l \leq 25$	$-11 \leq h \leq 12$<br>$-14 \leq k \leq 12$<br>$-20 \leq l \leq 20$	$-18 \leq h \leq 18$<br>$-21 \leq k \leq 21$<br>$-22 \leq l \leq 22$
Refl.collected/independent	29794 / 6458	26157 / 13779	29676 / 9950	14059 / 7372	48197 / 17137
R_{int}	0.0362	0.0305	0.0390	0.0309	0.0537
R_{sigma}	0.0311	0.0564	0.0494	0.0488	0.0676
Absorption correction	multi-scan	multi-scan	multi-scan	none	multi-scan
Max. and min. transmission	0.7465 and 0.5828	0.3812 and 0.3373	0.7465 and 0.6394	0.2986 and 0.2284	0.776 and 0.628
Refinement method	Full-matrix least-squares on F^2	Full-matrix least-squares on F^2	Full-matrix least-squares on F^2	Full-matrix least-squares on F^2	Full-matrix least-squares on F^2
Data/restraints/parameters	6458/0/198	13779/0/535	9950/0/316	7372/0/331	17137/0/746
Goodness-of-fit on F^2	1.038	1.020	1.034	1.053	1.021
Final R indices [I>2sigma(I)]	R1 = 0.0269, wR2 = 0.0635	R1 = 0.0378, wR2 = 0.0762	R1 = 0.0374, wR2 = 0.0746	R1 = 0.0384, wR2 = 0.0996	R1 = 0.0418, wR2 = 0.0796
R indices (all data)	R1 = 0.0368, wR2 = 0.0672	R1 = 0.0581, wR2 = 0.0831	R1 = 0.0544, wR2 = 0.0847	R1 = 0.0457, wR2 = 0.1043	R1 = 0.0684, wR2 = 0.0951
Larg. diff. peak/hole, $e·Å^{-3}$	0.479 and – 0.436	0.50/–0.480	0.559 and – 0.479	1.19/–0.78	0.49/–0.59
CCDC number	2345920	2346948	2345921	2346949	2347014
